# Sea urchin eggs contain a plastid-derived structure that contributes to their development

**DOI:** 10.1371/journal.pbio.3003705

**Published:** 2026-04-23

**Authors:** Tyler J. Carrier, Andrés Rufino-Navarro, Thorben Knoop, Urska Repnik, Andrés Mauricio Caraballo-Rodríguez, David M. Needham, Corinna Bang, Sören Franzenburg, Marc Bramkamp, Willi Rath, Arne Biastoch, José Carlos Hernández, Ute Hentschel

**Affiliations:** 1 GEOMAR Helmholtz Centre for Ocean Research, Kiel, Germany; 2 Zoological Institute, Christian-Albrechts University of Kiel, Kiel, Germany; 3 Departamento de Biología Animal, Edafología y Geología, Universidad de La Laguna, Tenerife, Canary Islands, Spain; 4 Federal Maritime and Hydrographic Agency, Hamburg, Germany; 5 Central Microscopy Facility, Christian-Albrechts University of Kiel, Kiel, Germany; 6 Skaggs School of Pharmacy and Pharmaceutical Sciences, University of California San Diego, San Diego, California, United States of America; 7 Collaborative Mass Spectrometry Innovation Center, Skaggs School of Pharmacy and Pharmaceutical Sciences, University of California San Diego, San Diego, California, United States of America; 8 Faculty of Mathematics and Natural Sciences, Christian-Albrechts University of Kiel, Kiel, Germany; 9 Institute of Clinical Molecular Biology, Christian-Albrechts University of Kiel, Kiel, Germany; 10 Institute for General Microbiology, Christian-Albrechts University of Kiel, Kiel, Germany; 11 Christian-Albrechts University of Kiel, Kiel, Germany; University of Georgia, UNITED STATES OF AMERICA

## Abstract

Development in the sea has long been thought to be a nutritional gamble that disproportionately ends in starvation. Here, we support the premise that components of plastids appear to be incorporated into sea urchin eggs and that these, in turn, benefit development. We find chromoplast-derived carotenoid crystals and chromoplast-specific metabolites inside the eggs of the sea urchin *Arbacia lixula*. We find evidence of plastid DNA in the eggs of 11 other sea urchins, with diatoms being the primary source and taxonomic richness of these plastid taxa directly related to egg size. The light-dependent activity of these chromoplast components influences phytohormone and lipid metabolism as well as offspring development, morphological plasticity, and survival. Offspring that benefit from these chromoplast components are predicted to disperse further, over larger geographic areas, and use a wider range of currents, including those that cross ocean basins. Data presented here challenge the long-held belief that components of non-metazoan organelles are unable to enter the germline and be passed between generations. We hypothesize that sea urchins manipulate plastids solely for their self-interest with the result of this process being a novel and adaptive form of maternal provisioning.

## Abstract 2

Development in the sea has long been thought to be a nutritional gamble that ends in starvation. This study shows that sea urchin eggs contain a plastid-derived structure and that the light-dependent activity of this plastid component influences phytohormone and lipid metabolism, as well as offspring development and survival.

## Introduction

The most common reproductive strategy amongst marine invertebrates involves producing a high number of small, energy-poor eggs [1,2]. These offspring develop into larvae that acquire nutrients by filtering particles (*e.g.*, phytoplankton, bacteria, and detritus) and taking up dissolved organics from the water column [3–5]. Availability of these nutritional resources varies considerably in coastal waters and are limited in the offshore waters where much of development takes place [6,7]. Weeks to months under these conditions can amount to extremely high mortality rates [8–10], and the few larvae that settle onto the seafloor have often dispersed 10s to 100s of kilometers along the continental slope or across an ocean basin [11,12]. This metabolic puzzle has led to the principle hypothesis that reproductive output counterbalances these ecological restrictions [1,2,13], but a complementary means to bridge this gap may be through microbial symbioses [14,15].

Animals have a long-standing developmental partnership with microbes [16], and marine invertebrates are no exception. Mothers use their reproductive machinery to transmit microbial symbionts that provide essential amino acids [17], contribute to nutritional plasticity [18], and hormonally regulate transitions between life stages [19]. Another feature of these communities is the presence of cyanobacteria [20,21], which commonly associate with the eggs, embryos, and larvae of sea urchins [14,15,20,22]. Photosymbiotic partnerships could, in principle, enable for long-distance dispersal in waters that are otherwise poorly equipped to support planktotrophic development [6,7,23]. Here, we test this hypothesis using *Arbacia lixula* (S1 Fig), a sea urchin that reproduces using an egg that is representative of planktotrohpy and whose larvae can disperse across the Atlantic Ocean from well-mixed populations in the Mediterranean Sea and Macaronesia [13,24].

## Results

### Presence of a plastid-derived structure

**Fig 1:**
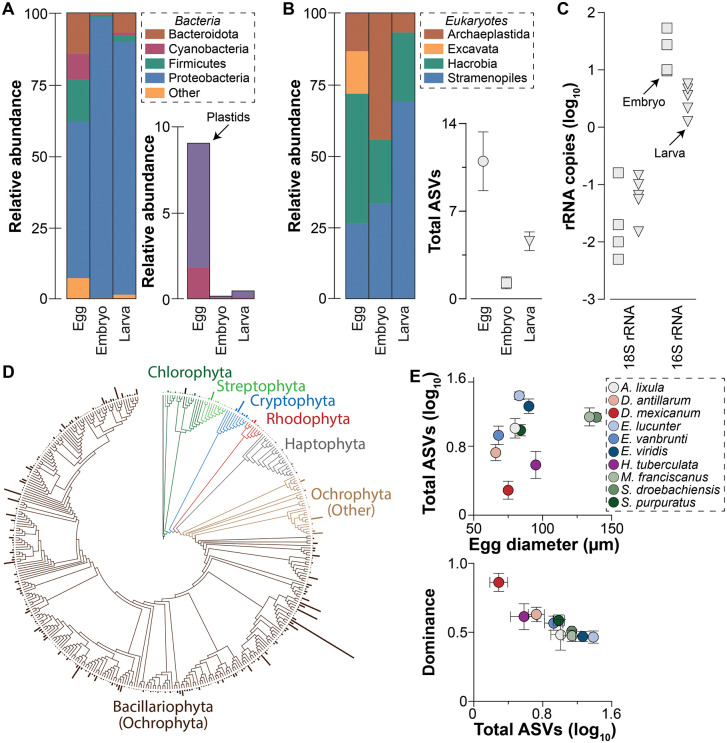
Plastid DNA in sea urchin eggs. **(A)** One of the main microbial phyla found to associate with the offspring of the sea urchin *Arbacia lixula* were Cyanobacteria (which do not contain plastids), but the majority of these sequences were unidentified plastids of photosynthetic eukaryotes. **(B)** All unidentified plastids could be traced to four major groups and less than a dozen ASVs of photosynthetic eukaryotes. **(C)** A substantial volume of *A. lixula* eggs would need to be allocated towards photosynthetic eukaryotes if their entire cell was maternally transmitted. A nuclear gene marker (18S rRNA gene) was disproportionately low in abundance in the metagenome of *A. lixula* embryos and larvae compared to a plastid gene marker (16S rRNA gene), implying that mothers may only provide her offspring with plastids. Corresponding raw data are presented in S1 and S8 Tables.

We compared the microbial communities of *A. lixula* eggs, embryos, and larvae (that were never fed) using amplicon sequencing of the 16S rRNA gene. These communities were primarily composed of the bacterial phyla Bacteroidota (7.3%), Cyanobacteria (3.4%), Firmicutes (5.8%), and Proteobacteria (80.5%) (Fig 1A). The majority (78.4% ± 5.0%) of cyanobacterial sequences associated with the egg were, however, unidentified plastid sequences that could be reassigned to photosynthetic eukaryotes (Fig 1A and 1B). These plastid sequences were derived from the Archaeplastida (13.3%), Excavata (14.9%), Hacrobia (45.3%), and Stramenopiles (26.5%) (Figs 1B and S2; S1 Table). We re-analyzed the egg-associated microbiota for a dozen other sea urchin species [22] and found an identical pattern: cyanobacteria (which do not contain plastids) and photosynthetic eukaryotes were both present, but photosynthetic eukaryotes represented nearly all of these sequences (S3 Fig; S2 and S3 Tables).

Plastid sequences were exclusive to and consistently present in the eggs of sea urchins that develop by planktotrohpy (*i.e.*, were not detected in non-feeding lecithotrophs; S4 Fig). These plastid taxa [*i.e.*, amplicon sequence variants (ASVs)] originated from eight eukaryotic phyla that fall within five kingdoms, with Ochrophyta being the most abundant and the Bacillariophyta (*i.e.*, diatoms) representing ~90.0% of these sequences (Figs 1D and S5; S4 and S5 Tables). Approximately 91.6% of Ochrophyta ASVs—and ~74.6% of all ASVs—were derived from diatoms (Figs 1D and S5; S4 and S5 Tables). Sea urchin eggs have a species-specific collection of plastid ASVs [Analysis of Similarity (ANOSIM), *p* < 0.001], which can more clearly be defined by geography (ANOSIM, *p* < 0.001) and time (ANOSIM, *p* < 0.001) (S6 and S7 Figs; S6 and S7 Tables). Richness of these plastid ASVs directly relates to egg size (linear regression: *F*_1,109_ = 7.80, *p* = 0.006, *R*^2^ = 0.067), whereby sea urchin species with smaller eggs tend to specialize on a few dominant ASVs while those with larger eggs had a relatively even distribution of a dozen or so ASVs (linear regression: *F*_1,109_ = 68.28, *p* < 0.0001, *R*^2^ = 0.385; Figs 1E, S8, and S9).

Associating with diverse photosynthetic eukaryotes with cell sizes similar to sea urchin eggs would present a geometric problem for the provisioning of macronutrients that are essential for early development (S10 Fig) [13,25]. An alternative strategy would be for mothers to provide her eggs with only the plastids from these photosynthetic eukaryotes. We used shotgun metagenomics to quantitatively compare a plastid (16S rRNA) and nuclear (18S rRNA) gene marker for photosynthetic eukaryotes. We found that each *A. lixula* embryo had 25.2 (± 20.7) copies of the 16S rRNA gene and 0.05 (± 0.07) copies of the 18S rRNA gene from photosynthetic eukaryotes (Figs 1C and S11; S8 Table). Notably, zero copies of the 18S rRNA gene were detected for most eukaryotic phyla, while copies of the 16S rRNA gene were consistently present (S11 Fig and S8 Table). Abundance of the 16S rRNA gene decreased significantly in larvae (7.3 × ; unpaired *t* test, *p* = 0.006), while the abundance of the 18S rRNA gene remained consistent (unpaired *t* test, *p* = 0.207) (Figs 1C and S4; S3 Table).

**Fig 2:**
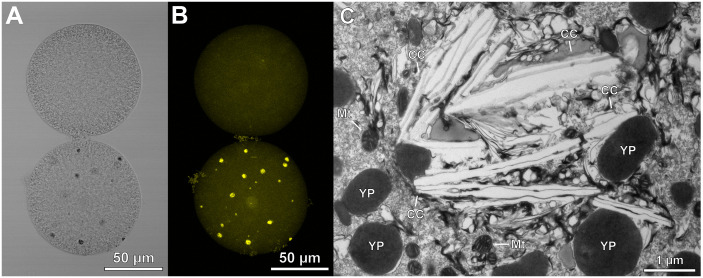
Presence of chromoplast-derived carotenoid crystal. **(A, B)** Micrographs of unfertilized eggs from the sea urchin *Arbacia lixula* without (top) and with (bottom) cytoplasmic autofluorescent particles. These particles were most consistent for the presence of carotenoids, as shown using (A) transmitted light and (B) fluorescence at an excitation of 561 nm. The light micrograph is a single optical slice, while the florescence micrograph is a maximum projection Z-stack. **(C)** These autofluorescent structures correspond with large crystals (CC) observed in transmission electron micrographs. Mitochondria (Mt) and yolk platelets (YP) are also noted. The combined fluorescence signature and cellular structure implies that these crystal structures are chromoplast-derived carotenoid crystals. Corresponding raw data are presented in S11 Table.

We fixed *A. lixula* eggs for fluorescence and transmission electron microscopy to determine whether plastids are present in *A. lixula* eggs [26]. We observed cytoplasmic structures with autofluorescence in most eggs, which lacked chlorophyll but were most consistent for the presence of carotenoids (Figs 2A, 2B, and S12). The ultrastructure of these particles were elongated crystals (Figs 2C and S13). The presence of plastid DNA and autofluorescent particles implies that these structures are chromoplast-derived carotenoid crystals (Figs 1 and 2). We could not identify chromoplast-specific starch granules and plastoglobules from similar structures that are commonly found in sea urchin eggs. Chromoplast-specific carotenoids (*e.g.*, xanthophylls) were, however, detected in the metabolome of *A. lixula* eggs (S9 and S10 Tables). The presence of multiple hallmark structures exclusive to chromoplasts [27,28] implies that a plastid-derived structure is present in these eggs.

### Benefits to offspring development

**Fig 3:**
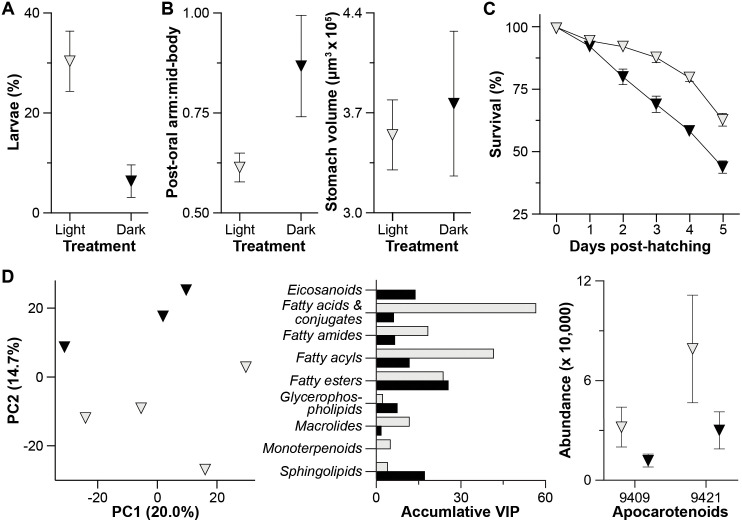
Benefits to offspring fitness. **(A)** More offspring of the sea urchin *Arbacia lixula* developed into 2-arm larvae in light (gray) than their siblings in dark (black) by 4 days post-fertilization. **(B)** Feeding arms of larvae that were cultured in dark grew disproportionately to the larval body (left), but did not exhibit a change in gut volume (right); thus, larvae in dark expressed the long-arm phenotype that corresponds with a nutritional limitation. **(C)** Survival was higher for offspring cultured in light, of which was also affected by time for both treatments as well as the interaction between these factors. Differences in light-induced survival was observed on days two through five. **(D)** Larvae cultured in light and dark exhibit organism-wide differences in their metabolome (left). This was predominantly driven by a shift in fatty acid metabolism, from the catabolism of energy storage lipids in light to structural lipids in dark (Variable Importance in Projection, VIPs; center). Two of the 717 differentially abundant metabolites are apocarotenoids, which were more abundant for larvae cultured in light (right).

Chromoplast-derived carotenoid crystals influence plant reproductive fitness and are involved in light-dependent reactions that are independent of photosynthesis [29,30]. Sea urchin development, on the other hand, is not known to be directly impacted by light. If these chromoplast-derived carotenoid crystal structures are beneficial to *A. lixula* offspring, then a “loss of function” (*i.e.*, darkness) should negatively impact development. We observed that *A. lixula* offspring undergo initial cell divisions more quickly in dark (*i.e.*, at 4 hpf; paired *t* test, *p* = 0.025), but have no observable difference upon gastrulation (paired *t* test, *p* = 1.000; S14 and S15 Figs; S11 Table). By four days post-fertilization, 4.8× more offspring developed into 2-arm larvae in light than their siblings in dark (paired *t* test, *p* = 0.026; Figs 3A and S14; S11 Table). The feeding arms of larvae that were cultured in dark grew disproportionately relative to the larval body (unpaired *t* test, *p* = 0.012; Figs 3B, S16, and S17; S11 Table); thus, larvae in dark expressed the long-arm phenotype that corresponds with a nutritional restriction [31]. However, unlike typical morphological plasticity, the gut volume did not differ between *A. lixula* larvae that were cultured in light and dark (unpaired *t* test, *p* = 0.952; Fig 3B and S11 Table) and that this occurred without any response to exogenous resources (*i.e.*, phytoplankton) [31].

A reduced developmental rate and modification to nutrition are two key factors that directly impact developmental fitness [8], which can be assessed by quantifying survival. We observed that the survival of *A. lixula* offspring was significantly reduced when cultured in dark, as well as with time and the interaction between these factors (two-way ANOVA, time: *F*_5,54_ = 143.6, *p* < 0.0001; treatment: *F*_1,54_ = 109.9, *p* = 0.0007, interaction: *F*_5,54_ = 10.31, *p* < 0.0001; Fig 3C and S11 Table). Differences in survivorship were observed two days post-hatching (*i.e.*, four days post-fertilization) and in all subsequent days (*p* < 0.001 for each day), such that survivorship was 1.4× higher in light at five days post-hatching (Fig 3C and S11 Table).

We then sought to understand how these chromoplast-derived carotenoid crystal structures impact the developmental metabolism of *A. lixula*. Light induced an organism-wide divergence in the metabolome during early development (PERMANOVA, eggs: *p* = 0.181, *p* < 0.001; Figs 3D and S18; S12 Table). This was predominantly driven by a shift in fatty acid metabolism, from the catabolism of energy storage lipids (*e.g.*, fatty acids, acyls, and esters) in light to structural lipids (*e.g.*, phospholipids and sphingolipids) in dark (Figs 3D and S19; S9, S10, and S13 Tables). Two of the 717 differentially abundant metabolites were apocarotenoids (*i.e.*, xanthophyll-derived phytohormones that promote growth and development, regulate lipid metabolism, and modulate environmental stress [32–34]). These apocarotenoids are a prenol lipid and an uncharacterized apocarotenoid, which were detected 2.7× more frequently in larvae in light and formed a molecular family with additional phytohormones and an energetic lipid (Figs 3D and S19; S9, S10, and S13 Tables). We, therefore, find that these chromoplast-derived carotenoid crystal structures and their derived metabolites have a broad and integral influence on the development of *A. lixula*.

### Enhanced and trans-oceanic dispersal

**Fig 4:**
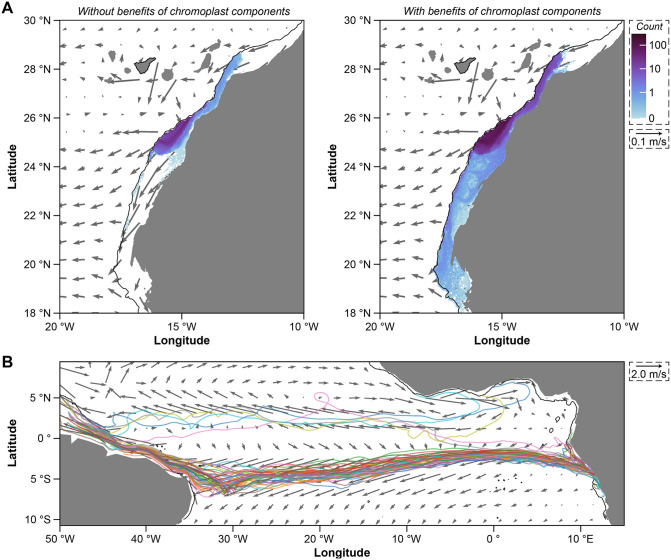
Enhanced and trans-oceanic dispersal. **(A)** Distribution of particles on the African shelf after 102 (*i.e.*, without benefits from the light-dependent activity of chromoplast components; left) and 181 (*i.e.*, with benefits from the light-dependent activity of chromoplast components; right) days of being released from Tenerife (black bordered island). Grey arrows show the 10-year mean velocity in the release depth from VIKING20X, with every 10th arrow is shown. Black contours around the African shelf mark the 500 m isobath. **(B)** Only particles that dispersed for 181 days from the southernmost point of *Arbacia lixula*’s range were predicted to disperse across the Northern Atlantic Ocean from the coast of Africa to Brazil. Fifty example trajectories are shown. Each plot is the accumulation of annual releases over 10 years. Grey arrows as in A, but only every 50th arrow is shown. Visualizations of larval dispersal used maps from cartopy [110], with the underlying vector map using NaturalEarth (naturalearthdata.com).

Development and survival can directly impact dispersal [1,2,9,10]. We used our survival data to estimate the potential pelagic larval duration for *A. lixula* that do (*i.e.*, light) and do not (*i.e.*, dark) benefit from the light-dependent activity of chromoplast components (Fig 3C). We estimated that the instantaneous mortality rate for *A. lixula* is 0.093 day^−1^ in light and 0.165 day^−1^ in dark, which are within the expected range for the marine invertebrates that develop by planktotrohpy and are higher than expected because the larvae in these experiments were never fed [8,9,35]. By quantifying the fecundity of *A. lixula* (S10 Fig), we then estimate that offspring that benefit from the light-dependent activity of chromoplast components have a 1.8× longer dispersal duration than those without it (S20 Fig). We then used the VIKING20X circulation model of the Atlantic Ocean to simulate dispersal [36]. We estimate that offspring that benefit from the light-dependent activity of chromoplast components have the potential to disperse 1.6× further (paired *t* test, *p* < 0.0001) and across a 4.7× larger geographical area (paired *t* test, *p* < 0.0001) than those without that benefit (Figs 4A and S21). A longer planktonic larval duration is estimated to enable 2.9× more larvae additional dispersal paths for a successful settlement (paired *t* test, *p* < 0.001; S21 and S24 Figs; S15 Table).

Offspring that benefit from the light-dependent activity of chromoplast components are also predicted to take fewer generations to spread across the longitudinal range of *A. lixula* (S25 Fig). At the southernmost point of this range, only larvae that benefit from the light-dependent activity of chromoplast components—an estimated 0.3% of the total—are predicted to disperse across the Atlantic Ocean (S26–S29 Figs). Nearly all of these larvae enter the Angola Current, merge with the South Equatorial Current, and then settle on the northern coast of Brazil (Figs 4B, S26, and S29). This east-to-west trans-Atlantic journey is successful each year despite the inter-annual variability in the strength of these currents (S28 Fig). Once on the northern coast of Brazil, offspring that benefit from the light-dependent activity of chromoplast components could disperse southward along the continental shelf, back to the African coast, and to Cape Verde (S30 Fig). It, however, would take 2.0× longer to reach the Azores and 4.3× longer to reach the Canary Islands than our estimated pelagic larval duration (S31 Fig and S15 Table).

## Discussion

Making a fertilizable egg is a complex and carefully regulated process that involves the provisioning of nutrients and maternal RNAs [37,38]. One long-held belief is that components of non-metazoan organelles (*e.g.*, plastids) are unable to cross the evolutionary valley between non-reproductive (somatic) and reproductive (germline) cells. Sea slugs [39] and flatworm [40] are the only animals known to retain stolen plastids from photosynthetic eukaryotes, yet any part of this highly stable organelle is not transmitted between generations. While vertical transmission was not shown explicitly, the data presented here provide initial evidence to question this assumption. Specifically, that eggs of the sea urchin *A. lixula* appear to have plastid components (*i.e.*, plastid DNA, chromoplast-derived carotenoid crystals, and chromoplast-specific metabolites) in their cytoplasm. Further supporting this hypothesis is that no other crystal structure has been reported in sea urchin eggs despite the cellular biology of fertilization and their pigments having been well-characterized [41,42] and that sea urchin eggs contain pigments and chloroplast-associated glycolipids that have been collectively referred to as the “chloroplast lipid” peak [43].

If chromoplast-derived carotenoid crystals are present in sea urchin eggs, then it remains paramount to understand the process that encompasses their isolation and incorporation. We hypothesize that this process—preliminarily termed “machiaplasty” for being characterized by the Machiavellian traits of manipulation, indifference, and self-interest—involves three stages. First, chloroplasts derived from diatoms—which are particularly stable and resistant to degradation, making them a common plastid source—and other photosynthetic eukaryotes are ingested by adult sea urchins while grazing [39,40,44]. Second, isolated chloroplasts differentiate into non-photosynthetic chromoplasts during the weeks to months spent inside the dark body cavity [27,28], a conversion that has been observed in plastids stolen by sea slugs [45]. Third, entire chromoplasts or, more likely, their specific components are then phagocytosed during oogenesis and utilized to partially fuel early development. We believe that this broad framework allows for the hypothesized process to be characterized (*e.g.*, by experimentally altering whether and which plastids are provided to adults) and for the direct impact of what we presume are chromoplast-derived carotenoid crystals on development to then be quantified (*e.g.*, by quantifying reproductive and developmental performance).

There is no indication that these chromoplast-derived carotenoid crystal structures are within an organelle inside the egg. There is, however, evidence supporting that phytohormones are more prevalent in larvae cultured in light and a light-dependent but photosynthesis-independent reaction that benefits development and survival. Light is expected to promote the swimming of these phototaxic larvae that, in turn, should utilize maternal energetics and result in the expression of the phenotype associated with nutrient limitation [46–48]. Light, or the lack thereof, is not known to directly influence the development, phenotype, or survival of marine invertebrate larvae that develop by planktotrohpy, as we have observed. Chromoplast-derived carotenoid crystals and their derived metabolites are involved in light-dependent reactions that promote development, regulate lipid metabolism, and are involved in protecting cells from oxidative stress and photo-damage [29,30,49]. Moreover, experimentally enriching xanthophylls in the diet of adult sea urchins can enhance offspring size, developmental rate, and survival [50]. We, thus, presume that these plastid components regulate lipid metabolism that, in turn, impacts the development and survival of *A. lixula*. Benefits to the offspring without a distinct benefit to the adults should, in turn, have a net positive benefit across the entire host’s lifecycle, which may result in this being an adaptive process [51].

The incorporation of chromoplast-derived carotenoid crystals should increase the total resources provisioned into an egg and, in turn, produce more fit offspring. This additional maternal resource enhances dispersal potential, which is most pronounced through the prediction that only offspring that benefit from the light-dependent activity of these chromoplast components have the trajectory to potentially undergo trans-Atlantic dispersal. While exogenous resources are required for planktotrophic larvae to develop, the metabolic benefit of these plastid components is predicted to increase, but not exclusively support, the dispersal trajectory of these larvae. This, in turn, could provide offspring with an elevated potential to cross an ocean basin and to use this major oceanic current as a highway for gene flow [12,24,52]. Providing offspring with plastid-derived components in the sunlit ocean could, thus, serve as a mechanism to influence long-distance (*i.e.*, teleplanic) dispersal. This developmental feature is observed for most groups of marine invertebrates and in every major ocean, including sea urchin larvae that use the South Equatorial Current to cross the Atlantic Ocean [12,52]. These plastid-derived components are hypothesized to be a novel form of maternal provisioning and a complementary metabolic resource to the nutrients acquired by filtering particles, taking up of dissolved organics, or through microbial symbioses [3–5,17].

## Materials and methods

### Specimen collection

Adult *Arbacia lixula* were collected by snorkel from Radazul Beach (Tenerife, Canary Islands, Spain; 28.401487, −16.324368) from 2021 to 2024. Adult sea urchins were transferred dry to the University of La Laguna within one hour, where they were maintained in an aerated aquarium with seawater from Radazul Beach until spawning a couple hours later.

### Offspring culturing

Gravid adults were spawned by an intracoelomic injection of 0.50 M KCl. Females were rinsed with 0.22 μm FSW to remove potential contamination by epibionts on the aboral surface—despite it being widely acknowledged that epibionts are unable to colonize sea urchin spines [53,54]—and then spawned into 5.0 μm filtered seawater (FSW) from Radazul Beach, while males were spawned dry and stored on ice. Eggs were subsequently rinsed three times with 0.22 μm FSW to remove potential contamination during spawning. Female and male gametes were collected to generate full-sibling replicates (*n* = 5) by following Strathmann [55].

Briefly, a few drops of diluted (1:1,000) sperm from the male pair was added to the total clutch of the female pair. Egg and sperm were gently mixed. Fertilization was assessed 30 min later using a compound microscope (Leica DM1000 with a Canon EOS 1200D camera). Cultures were diluted in 5.0 μm FSW (that allows developing embryos to be colonized by their natural microbiota) to 2 embryos/mL and each male-female pair was divided amongst separate light and dark beakers. Dark beakers were covered with multiple layers of tinfoil, equipped with a cardboard lid that was also covered with multiple layers of tinfoil and an overhang where the lid and beaker meet, and a loose-fit black garbage bag on the stirring paddle to exclude all possible light from entering. All beakers were stirred gently and ~95% of the FSW was replenished every other day with 5.0 μm FSW. Offspring in the dark beakers were minimally exposed to light during mandatory husbandry (*i.e.*,~5 min per water change). Eggs, embryos, and larvae were cultured in ambient FSW at 23 °C (*i.e.*, ambient sea surface temperature during autumn in Canary Islands coastal waters [56]), provided a combination of ambient solar light and artificial laboratory light (photosynthetically active radiation of ~570.3 ± 100.0 μmol photons m^−2^ s^−1^ to reflect light incidence with alternate cloudy and sunny days in Canary Islands [57]), and never provided phytoplankton or another external dietary source.

### Sample collection, extraction, and sequencing

We collected eggs (0 hours post-fertilization, hpf), embryos (blastula; 18 hpf), and early-stage larvae (4 days post-fertilization, dpf) from each “light” beaker (*n* = 5) for amplicon and metagenomic sequencing; both sequencing types were from the same sample. Approximately 200 offspring were collected from each male-female pair at each of the developmental stages. Offspring were concentrated using a microcentrifuge, the seawater was removed with a sterile pipette, and tissues were preserved in 250 µL of 70% ethanol for long-term storage at −20 °C. Samples were then transferred on blue ice to the GEOMAR Helmholtz Centre for Ocean Research (Kiel, Germany).

Total DNA was extracted from all samples and DNA kit blanks (*n* = 3) according to the manufacture’s protocol for the DNeasy Blood & Tissue Mini Kit (Qiagen). Total DNA was then quantified using the dsDNA HS Assay Kits for the Qubit Fluorometer (Thermo Fisher Scientific) following the manufacture’s protocol. Samples were diluted to 0.5 ng/µL for amplicon sequencing and were not diluted for metagenomic sequencing. Samples for amplicon (V3/V4 region of the 16S rRNA gene) and metagenomics were sent to the Competence Center for Genomic Analysis (Christian-Albrechts University of Kiel, Germany) for library preparation and sequencing by Illumina MiSeq (v3, 2 × 300 bp paired-end reads) and Illumina NovaSeq (S4, 2 × 150 bp paired-end reads), respectively.

### Amplicon analysis of *A. lixula*

Raw amplicon reads with quality information were imported into QIIME 2 (v. 2022.11; [58]), where forward and reverse reads were paired using VSEARCH [59], filtered by quality score, and denoised using Deblur [60]. QIIME 2-generated “features” were analyzed as ASVs [61] and were assigned taxonomy using SILVA (v. 138; [62]). Sequences matching to Archaea, those that were disproportionately present in the DNA kit blanks, and samples with less than 3,000 reads were discarded. The filtered data table was then rarified to 3,482 sequences (S32 Fig and S16 Table). We summarized taxonomic groups of bacteria for each sample from the SILVA [62] assignment. We then filtered this data table and used PhytoREF [63]—a database for plastidial 16S rRNA gene sequences of photosynthetic eukaryotes—to re-assign taxonomy for all ASVs that were previously assigned to “o__Chloroplast.” It is worth noting that PhytoREF uses outdated taxonomic information for Eukaryotes [64,65], which may limit taxonomic accuracy at the finer levels. Taxonomic overlap in the plastid ASVs that were present in the eggs of each female *A. lixula* was determined using InteractiVenn [66].

### Amplicon meta-analysis of sea urchins

We compiled all published 16S rRNA datasets for sea urchin eggs (S2 Table) [15,22,67,68], as well as single samples of *Eucidaris thouarsii* and *Eu. tribuloides* that was collected during, but not published with, Carrier *and colleagues* [15]. We generated taxonomic profiles for each sea urchin species and then tallied the relative abundance of “o__Chloroplast” to the non-plastid Cyanobacteria sequences, and then reassigned the taxonomy for “o__Chloroplast” using PhytoREF [63]. We transformed the plastid-only data table to relative frequency to avoid discarding invaluable sequences. Using this, we generated a phylogenetic tree for all plastid ASVs within QIIME 2 [58] using MAFFT [69] and FastTree [70]. This tree was then visualized using the Interactive Tree Of Life [71] and stylized using Adobe Illustrator (v. 24.0.1). Mean relative frequency of each plastid ASV was added as metadata to the phylogenetic tree. Next, we manually curated the prevalence of plastids in each sample and ASVs across species. We then calculated Jaccard distances for all samples, visualized these using principal coordinate analyses in Prism (v. 9.0.0), and stylized these using Adobe Illustrator. ANOSIM and PERMDISP, as well as their respective pairwise comparisons, were performed within QIIME 2 [58] to test whether community composition and dispersion varied between host species, geography, and years.

Jaccard distances were then used to construct a dendrogram within QIIME 2 [58]. The data table for this analysis was collapsed by host species and then was rarified to 447 sequences; this was the only rarified analysis. We then tested for phylosymbiosis by comparing topological congruence with a Cytochrome C Oxidase Subunit I (COI) gene tree using sequences from the National Center for Biotechnology Information (NCBI) (S2 Table). These sequences were imported into the Molecular Evolutionary Genetics Analysis software (v. 11.0.9; [72]), aligned with MUSCLE [73], and trimmed. This relationship was inferred using maximum likelihood with the optimized DNA substitution model (GTR + G + I, as determined by BIC criteria) and 1,000 bootstrap replicates. Phylosymbiosis was tested using the Robinson-Foulds metric in TreeCmp (v. 2.0; [74]) and matching cluster metrics with 10,000 random trees [75].

Four measures of alpha diversity (total ASVs, Faith’s phylogenetic distance, McIntosh evenness, and McIntosh dominance) were then calculated for all samples. These were compared across species using one-way ANOVAs and Tukey’s post-hoc tests for pairwise comparisons. Linear regression analysis was performed to compare total plastid ASVs for each sea urchin species to published average values of egg diameter [13,76–78], total energetic content [77,79–81], and pelagic larval duration [76,82–87] for these same sea urchin species in Prism. A linear regression analysis was also used to compare total plastid ASVs and McIntosh dominance in Prism.

### Metagenomic analysis

Raw metagenomic reads were quality filtered, trimmed, and removed of host sequences within MetaWRAP [88]. The *S. purpuratus* genome (v. 5.0) from Echinobase [89,90] was used to minimize host reads because there was no published genome for *A. lixula* at the time of this analysis. Furthermore, we removed mitochondrial sequences using published mitochondrial genomes for *A. lixula* (NC_001770), *Echinometra mathaei* (NC_034767), *Hemicentrotus pulcherrimus* (NC_023771), *Heterocentrotus mammillatus* (NC_034768), and *Lytechinus variegatus* (NC_037785) from NCBI.

rRNA gene sequences were identified in the forward sequences using METAXA2 [91]. All rRNA gene sequences were imported into QIIME 2 [58] for taxonomic assignment using the default parameters of the VSEARCH [59] consensus classifier. Plastid sequences (*i.e.*, 16S rRNA gene) were identified using PhytoREF, while nuclear sequences (*i.e.*, 18S rRNA gene) were identified using the combined Protist Ribosomal Reference (PR^2^) [92]/EukRef [93] 18S rRNA databases. The quantity of 16S and 18S rRNA gene sequences were counted for all equivalent groups of photosynthetic eukaryotes (S8 Table). Sequences for 16S and 18S rRNA gene were then summed separately and compared between developmental stages using unpaired two-tailed *t*-tests within Prism.

### Microscopy

Fluorescence microscopy was used to assess whether there were autofluorescent signatures consistent with plastid pigments in unfertilized eggs of *A. lixula*. Eggs (*n* = 10) were fixed at room temperature in 4% formaldehyde buffered in ambient seawater supplemented with 20 mM HEPES. This fixative was replaced after 1 hour with 1% formaldehyde buffered in ambient seawater with 20 mM HEPES buffer, before being stored long-term at 4 °C. Eggs were assessed for autofluorescence above 620 nm at excitation wavelengths of 405 nm, 488 nm, 561 nm, and 640 nm using an LSM 900 with Airyscan microscope (Zeiss) in the Multiplex mode. Maximum intensity projections of Z-stacks were generated using the ZEN Blue (v. 3.2) software (Zeiss). Transmitted light images at single Z-planes were acquired with the LSM mode. A Plan-Apochromat 20× (0.8 NA) objective was used for all applications.

Transmission electron microscopy was used to assess the ultrastructure of unfertilized eggs of *A. lixula* from the same females (*n* = 10) that were sampled for fluorescence microscopy. Eggs were fixed with 0.5% glutaraldehyde buffered in ambient seawater for 1 hour and then stored long-term at 4 °C [26]. For resin embedding, the eggs were washed with deionized water and glycine to remove the fixative, post-fixed with 1% osmium tetroxide in 1.5% potassium ferricyanide for 1 hour at room temperature, washed several times with deionized water, treated with 0.1% tannic acid in 50 mM HEPES (pH 7) for 3 min at room temperature, stained with 2% aqueous uranyl acetate for 1 hour, and then washed again with deionized water. Samples were dehydrated using a graded ethanol series, followed by 100% acetone, and then progressively infiltrated with Epon 812 substitute resin (Sigma-Aldrich) and heat polymerized. Ultrathin 80-nm sections, prepared using the UC7 ultramicrotome (Leica) and diamond knives (Diatome), were contrasted with saturated aqueous uranyl acetate and lead citrate. Grids were inspected in a Tecnai G2 Spirit BioTWIN transmission electron microscope using a side-entry CCD MegaView III G2 camera and iTEM software (v. 5.0) (Olympus Soft Imaging Solutions).

### Development, morphology, and survival

We measured egg size, development, and morphology as part of the experiment outlined in Sample collection, extraction, and sequencing section. Egg diameter (*n* = 10 per replicate) was measured at 0 hpf for all “light” beakers, while the developmental stage (*n* = 25 per replicate per time point) at 0 hpf, 4 hpf, 18 hpf, 2 dpf, and 4 dpf was assessed for all beakers. Offspring were staged as either egg, 2-cell embryo, 4-cell embryo, 8-cell embryo, 16-cell embryo, blastula, gastrula, prism-stage larva, or 2-arm larva. We quantified average cell number per embryo at 4 hpf and frequency of 2-arm larva at 4 dpf to assess developmental rate, which were both compared statistically by a *t* test. Morphometrics (*i.e.*, mid-body line, post-oral arms, stomach height, and stomach width) were performed on all 2-arm larva at 4 dpf using ImageJ (v. 1.9.2; [94]). Due to reduced development and survival in dark, 2-arm larvae from light and dark replicates (*i.e.*, *n* = 49 in light and *n* = 10 in dark) were merged and then compared statistically within Prism using an unpaired two-tailed *t* test for each morphological measurement.

Survivorship was quantified in separate experiments using the exact same experimental design. Offspring from male-female pairs (*n* = 10) developed until hatching (*i.e.*, 2 dpf), after which triplicate counts of each culture density was performed and were subsequently diluted to 2 embryos/mL in 1 L of FSW. Cultures were reversed-filtered to 100 mL each day, the number of embryos were counted in triplicate 1 mL aliquots using a dissecting microscope (Leica EZ4), and then returned to the beaker along with fresh FSW. This was performed for five days (*i.e.,* until 7 dpf). Five of the biological replicates were performed in 0.22 μm FSW and the other five were performed in 5.0 μm FSW as a cross-validation for the agent being intracellular. Both survival datasets were merged because the agent was intracellular (Fig 2) and because the survival rates were statistically identical between both conditions (two-way ANOVA for light, time: *F*_5,40_ = 102.8, *p* < 0.0001; treatment: *F*_1 8_ = 0.04, = , interaction: *F*_5,40_ = 3.91, *p* < 0.0055; two-way ANOVA for dark, time: *F*_5,40_ = 93.33, *p* < 0.0001; treatment: *F*_1 8_ = 0.38, *p* = 0.554, interaction: *F*_5,40_ = 1.06, *p* = 0.399; S11 Table). A two-way ANOVA was performed within Prism to test whether survivorship differed between treatments and over time/development.

### Metabolomics

Metabolomics was used in a separate experiment using the exact same experimental design as development and survival. Specifically, the clutch of spawned females (*n* = 5) was equally distributed between separate 1 L beakers for light and dark. Three technical replicates of ~1,000 eggs were collected from all beakers, which were immediately centrifuged to concentrate the tissues, the 0.22 μm FSW was removed, samples were flash frozen using liquid nitrogen, and then transferred to a −80 °C freezer for long-term storage. The remaining eggs were fertilized with dilute sperm to create full-sibling replicates. Excess sperm was removed 1 hour later by multiple 95% water changes and fertilized embryo were then provided two days to develop. Hatched blastulae were isolated, the cultures were diluted to 10 embryos/mL following multiple 95% water changes, and the cultures were then provided 3 additional days to develop (*i.e.*, when there was a maximal differential in survival; Fig 3C). At 5 dpf, three technical replicates of ~1,000 offspring were collected from all beakers and processed as described above. Four and three biological replicates could be collected from the light and dark treatments of larvae, respectively. Blank tubes and 0.22 μm FSW seawater were also collected as controls (*n* = 5 each). All samples were then shipped on dry ice (with temperature being monitored) to the University of California, San Diego (CA, USA).

Samples were prepared for liquid chromatography tandem mass spectrometry (LC–MS/MS) according to an established protocol [95]. Untargeted LC–MS/MS was then performed using a Vanquish Flex Ultra-High-Performance Liquid Chromatography (UHPLC) system that was coupled with an Orbitrap Exploris 240 Mass Spectrometer (Thermo Fisher Scientific). Chromatographic separation was performed using a Kinetex 2.6 µm Polar C18 reversed phase UHPLC column with a constant flow rate of 500 μL/min (Phenomenex). LC–MS/MS acquisition followed an isocratic elution and a multistep linear gradient (S14 Table). Data-dependent acquisition mode was then used for acquisition of tandem MS (S14 Table). Analytical blanks, pool samples, and mixtures of sulfamethazine, sulfamethizole, sulfachloropyridazine, sulfadimethoxine, amitriptyline, and coumarin-314 at 10 μM were injected after every 10 samples as quality control for monitoring instrument performance.

Raw LC–MS/MS data files were processed in MZmine (v. 4.6.1; [96]) to generate a feature table, peak list, and batch file of all settings, with peak area of detected ions being used as metabolite abundance (S10 Table). Annotations and chemical classification of detected molecules were obtained with SIRIUS (v. 6.1.1.1; [97]) (S9 Table), with these annotations corresponding to level 3 of the Metabolomics Standards Initiative [98,99]. This was then used for a feature-based molecular network [100] in GNPS2 [101]. We generated a rarefaction curve to assess whether the metabolite diversity is fully represented (S33 Fig). We performed a principal component analysis and separate PERMANOVA in R to compare the metabolome across developmental stages as well as between light and dark for eggs and larvae. We then performed a Partial Least Squares Discriminant Analysis to identify differentially abundant metabolites (*i.e.*, Variable Importance in Projection, or VIPs) of larvae in light and dark. We summarized the total contribution of these differentially abundant metabolites in light and dark at the pathway, superclass, and class levels. Lastly, we used normalized data to summarize the abundance of two VIPs and assessed which metabolites are within their respective molecular family from the feature-based molecular network [102].

### Estimating and modeling dispersal

We calculated the duration required for the survival of the last larva from the clutch of *A. lixula* with and without the light-dependent activity of what we presume are chromoplast components. The abundance of *A. lixula* offspring (*N*_*t*_) after a specific time interval (*t*; days) can be calculated using the instantaneous rate model: *N*_*t*_ = *N*_0_ *e*^−*Mt*^, where *N*_0_ is the fecundity, *e* is the Naperian constant, and *M* is the instantaneous rate of mortality (day^−1^) [8]. *M* was calculated using the survival data for *A. lixula* offspring in light and dark. Fecundity (*N*_0_) was estimated by spawning individuals (*n* = 5) until eggs were no longer released, standardizing by total volume, performing two subsequent 1:100 dilutions for 1 mL fully concentrated eggs, and counting triplicate 0.5 mL aliquots of dilute eggs using a dissecting microscope (Leica EZ4). The specific time interval was then calculated for offspring that do and do not benefit from the light-dependent activity of chromoplast components. We compared our instantaneous rates of mortality to the literature to verify if larvae cultured in light and dark were both well within the expected range for the marine invertebrate larvae that develop by planktotrophy [8,9,35].

We integrated these specific time intervals into the VIKING20X [36], a full-scale, nested, and eddy-rich oceanographic model of the Atlantic Ocean. Specifically, we used the VIKING20X-OMIP [36] to perform our dispersal experiments because of its realistic ability to simulate atmospheric forcing [103], to accurately simulate the large-scale horizontal circulation, distribution of mesoscale, overflow, and convective processes, and the representation of regional current systems in the North and South Atlantic Ocean [104,105]. This combination of oceanographic features has previously enabled the VIKING20X to be used to simulate larval dispersal of coastal and deep-sea marine invertebrates [106,107]. We used the Lagrangian simulation software tool Parcels (v. 2.3.1; [108]) to simulate the 3D advection of virtual larvae based on the daily-mean velocities for ocean currents in the upper 12 vertical levels (~0–130 m) from 2007 to 2016. Virtual particles were released at depths between 0 and 30 m in the coastal waters around Tenerife (S21 Fig). Our simulation released 10,000 particles each week for 10 years (*i.e.*, from 2007 to 2016) to account for seasonal and inter-annual variability. Virtual particles had a lifetime of either 102 (*i.e.*, without benefits from the light-dependent activity of chromoplast components) or 181 days (*i.e.*, with benefits from the light-dependent activity of chromoplast components). The abundance, geographical location, and distance of particles from each treatment that reached the continental shelf of Africa were tracked. Particles that reached the continental shelf of Africa in 25 days or less were not counted due to the minimal pelagic larval duration for *A. lixula* [78]. Each of these quantitative assessments were compared statistically using paired two-tailed t-tests within Prism.

Using this same oceanographic framework, we determined how many iterations (*i.e.*, generations) it took for virtual larvae to spread along the African shelf and reach the southernmost range for *A. lixula*. Generation 0 was considered Tenerife and Generation 1 was the resulting dispersal. The location and abundance of particles from Generation 1 were then used to determine the origins and then spread in Generation 2. This was repeated until a particle reached the southernmost range for *A. lixula*. Next, we evenly distributed 2.0 million particles along the African shelf (from 28°N to 10°S; S25 Fig) to determine if any and which locations could be used as the departure area for trans-Atlantic dispersal. Only particles with a pelagic dispersal potential reflecting the benefits from the light-dependent activity of chromoplast components and initially located at the southernmost area could disperse across the Atlantic Ocean (Fig 4B), so we then tracked paths that these particles took and compared them to known oceanographic currents. Lastly, we evenly distributed 1.6 million particles along the northern coast of Brazil (from 5°S to 10°S; S30 Fig) to determine if larvae that benefit from the light-dependent activity of chromoplast components could disperse back to Macaronesia and towards Uruguay (*i.e.*, the southernmost range for *A. lixula* in South America) [24,109]. This modeling experiment was performed for four years to determine when virtual particles reached the Azores and the Canary Islands. Visualizations of larval dispersal used maps from cartopy [110], with the underlying vector map using NaturalEarth (naturalearthdata.com).

## Supporting information

S1 Fig Experimental system. An adult (left) and larva (right) of the sea urchin *Arbacia lixula*.
(TIF)

S2 Fig Plastid ASVs in sea urchin eggs. Minimal taxonomic overlap **(A)** and the diversity **(B)** of plastid ASVs in egg from different individuals of the sea urchin *Arbacia lixula*. Empty cells in the Venn diagram have zero ASVs. Corresponding raw data are presented in S1 Table.
(TIF)

S3 Fig Relative abundance of plastids and cyanobacteria. Abundance of plastid sequences from photosynthetic eukaryotes relative to that of cyanobacteria in the egg-associated microbiota of the 12 sea urchins that have been studied to date. Note: *Heliocidaris erythrogramma* that is present in S3 Fig was not included here because these taxonomic groups are not present. See S2 Table for replication. Corresponding raw data are presented in S3 Table.
(TIF)

S4 Fig Prevalence of plastid ASVs in sea urchin eggs. Plastid ASVs were present in the eggs of all but one sea urchin species, which was the only sea urchin that develops and undergoes metamorphosis without the requirement of external nutrients through feeding. The other species develop via feeding larvae, of which plastid DNA was present in 91% of these samples. Note: the two *Eucidaris* species in S2 Fig were not included in the rest of this meta-analysis because the single sample for each is insufficient for a full comparison. See S2 Table for replication and S3 Table for the corresponding raw data.
(TIF)

S5 Fig Taxonomic profile of plastid ASVs. Taxonomic profile of the plastid ASVs from photosynthetic eukaryotes in sea urchin eggs. All groups represent eukaryotic phyla except the Bacillariophyta (diatoms), which are presented at the class-level and is part of the Ochrophtya. See S2 Table for replication and S4 Table for the corresponding raw data.
(TIF)

S6 Fig Species and location-specificity in plastid ASVs. **(A)** Principal coordinate analysis depicting community relatedness (Jaccard) of the plastid ASVs in sea urchin eggs. Color and shape denote species and geographic locations, respectively. Square, circle, triangle, and hexagon represent Friday Harbor (WA, USA), Panama City and Colón (Panama), Sydney (Australia), and Tenerife (Canary Islands, Spain), respectively. **(B)** Topology of the host gene tree (using COI) is congruent with a dendrogram for these plastid ASVs based on species, even though they do not fully mirror each other.
(TIF)

S7 Fig Temporal differences in plastid ASVs. Principal coordinate analysis depicting community relatedness (Jaccard) of plastid ASVs that are associated with the eggs of the confamilials *Strongylocentrotus purpuratus* (triangle), *Mesocentrotus franciscanus* (square), and *S. droebachiensis* (circle) that were collected in 2016 (blue) and 2019 (pink) in Friday Harbor (WA, USA).
(TIF)

S8 Fig Plastid diversity in sea urchin eggs. This was estimated by total ASVs, Faith’s phylogenetic diversity, McIntosh dominance, and McIntosh evenness. Each circle species average ± standard error. Corresponding raw data are presented in S6 Table.
(TIF)

S9 Fig Egg size not related to life-history traits. No trade-off was observed between the energetic content (C; mJ; linear regression: *F*_1,109_ = 1.36, *p* = 0.246, *R*^2^ = 0.012) of sea urchin eggs or pelagic larval duration (D; days; linear regression: *F*_1,109_ = 0.380, *p* = 0.539, *R*^2^ = 0.003) and the diversity of plastid ASVs that were provided to sea urchin eggs. Each circle represent a species average with their corresponding standard error bar. Corresponding raw data are presented in S6 Table.
(TIF)

S10 Fig Egg size and fecundity. Average egg size (diameter; µm) **(A)** and fecundity **(B)** for five individuals of the sea urchin *Arbacia lixula*. Corresponding raw data are presented in S11 Table.
(TIF)

S11 Fig Lack of a nuclear gene for most photosynthetic eukaryotes. A nuclear gene marker (18S rRNA gene) was disproportionately low in abundance or not present in embryos of the sea urchin *Arbacia lixula* compared to a plastid gene marker (16S rRNA gene) for the Alveolata (Dinophyta), Archaeplastida (Chlorophyta, Glaucophyta, Rhodophyta, Streptophyta), Excavata (Discoba), Hacrobia (Cryptophyta, Haptophyta), and Stramenopiles (Ochrophyta). This is a group-by-group display of the rRNA counts presented in Fig 1C, with corresponding raw data being presented in S8 Table.
(TIF)

S12 Fig Cytoplasmic autofluorescent structure in eggs. Light micrographs of unfertilized eggs from the sea urchin *Arbacia lixula* without (top) and with (bottom) cytoplasmic autofluorescent particles, as displayed by transmitted light **(A)** as well as at an excitation of 405 nm **(B)**, 488 nm **(C)**, 561 nm **(D)**, and 640 nm **(E)**. The light micrograph is a single optical slice, while the florescence micrograph is a maximum intensity projection of a Z-stack. Note: A and D are identical to Fig 2A and 2B, respectively. These are displayed here again to allow for direct comparison across wavelengths.
(TIF)

S13 Fig Diversity of chromoplast-derived carotenoid crystals. Transmission electron micrographs of unfertilized eggs from the sea urchin *Arbacia lixula* with what we presume are large tetragonal carotenoid crystals (CC) that are derived from chromoplasts. Chromoplast-specific starch granules and plastoglobules could not be distinguished within *A. lixula* eggs. Also in view are yolk platelets (YP) and mitochondria (Mt) that are presumed to be maternal.
(TIF)

S14 Fig Summary of developmental profiles. The developmental stages for offspring of the sea urchin *Arbacia lixula* were recorded at several intervals while being cultured from fertilization through four days post-fertilization in light (*i.e.*, with benefits from the light-dependent activity of chromoplast-derived components) and dark (*i.e.*, without benefits from the light-dependent activity of chromoplast-derived components). Corresponding raw data are presented in S11 Table.
(TIF)

S15 Fig Increase in cell division. Offspring develop quicker in dark (*i.e.*, without benefits from the light-dependent activity of chromoplast-derived components) during the first four cell divisions (*i.e.*, from fertilization to the 16-cell stage), as compared to their siblings in light (*i.e.*, with benefits from the light-dependent activity of chromoplast-derived components). All values are average ± standard error. These data are from 4 hours post-fertilization (S14 Fig). Corresponding raw data are presented in S11 Table.
(TIF)

S16 Fig Cartoon larva. Schematic of the morphological features that were measured to assess whether the two-arm larvae (four days post-fertilization) of the sea urchin *Arbacia lixula* exhibited morphological plasticity.
(TIF)

S17 Fig Light-induced developmental plasticity for some morphological features. The length of the post-oral arms **(A)** and the mid-body line **(B)** were similar between treatments for larvae of the sea urchin *Arbacia lixula* that were cultured in light (*i.e.*, with benefits from the light-dependent activity of chromoplast-derived components), as compared to their siblings in dark (*i.e.*, without benefits from the light-dependent activity of chromoplast-derived components). All values are average ± standard error (*i.e.*, the combination across all larvae from light or dark replicates; *n* = 49 in light and *n* = 10 in dark). Corresponding raw data are presented in S11 Table.
(TIF)

S18 Fig Light-induced metabolic divergence during development. Eggs of the sea urchin *Arbacia lixula* that were placed in light (*i.e.*, with benefits from the light-dependent activity of chromoplast-derived components) and dark (*i.e.*, without benefits from the light-dependent activity of chromoplast-derived components) had similar metabolomes (left and center). A metabolome-wide shift occurred during development (left) and based on the presence of light for larvae (right). These PCA plots are displayed with each technical replicate **(A)** as well as the median of those technical replicates **(B)**. Larvae with the median of their technical replicates is Fig 3D.
(TIF)

S19 Fig Differentially abundant metabolites and apocarotenoid modules. **(A)** Larvae cultured in light and dark exhibit organism-wide differences in their metabolism. **(B)** This was predominantly driven by a shift in fatty acid metabolism, including dicarboxylic acids as well as several other complementary fatty acids that were mainly differentially abundant in light. **(C)** Metabolite 9409 (a prenol lipid) was part of a metabolic module that had three other known metabolites that were found in the offspring of the sea urchin *Arbacia lixula*. This module included two uncharacterized apocarotenoids and jasmonic acid, all of which are phytohormones. **(D)** Metabolite 9421 was part of a metabolic module that included a prenol lipid and fatty acyl. Corresponding raw data are presented in S9, S10, and S13 Tables.
(TIF)

S20 Fig Enhanced planktonic larval duration. Planktonic larval duration was estimated for development in light (*i.e.*, with benefits from the light-dependent activity of chromoplast components) and dark (*i.e.*, without benefits from the light-dependent activity of chromoplast components) using the fecundity of the sea urchin *Arbacia lixula* and the instantaneous rate model for the natural mortality of marine invertebrate larvae. It is estimated to take 181 and 102 days in light and dark, respectively, for the last larva from an entire clutch of an individual *A. lixula* to remain. Visualizations of larval dispersal used maps from cartopy [110], with the underlying vector map using NaturalEarth (naturalearthdata.com). Corresponding raw data are presented in S11 Table.
(TIF)

S21 Fig Enhanced dispersal. Offspring cultured in light (*i.e.*, with benefits from the light-dependent activity of chromoplast components) enhances the total dispersal distance **(A)**, geographical area that is settled on **(B)**, and abundance of particles reaching a habitat to settle **(C)**, as compared to their siblings in dark (*i.e.*, without benefits from the light-dependent activity of chromoplast components). These numerical values were extracted from Fig 4A, of which are derived from the distribution of particles on the African shelf after 102 (*i.e.*, without benefits from the light-dependent activity of chromoplast components) and 181 (*i.e.*, with benefits from the light-dependent activity of chromoplast components) days of being released from Tenerife (Canary Islands, Spain). All data were log-transformed for normality. Each dot represents the 10-year average ± standard error. Visualizations of larval dispersal used maps from cartopy [110], with the underlying vector map using NaturalEarth (naturalearthdata.com). Corresponding raw data are presented in S15 Table.
(TIF)

S22 Fig Location for particle release. Location of particle release from Tenerife (Canary Islands, Spain) at the grid resolution of the VIKING20X model. Particles were released from grid cells that were <500 m (red squares) adjacent to Tenerife. Visualizations of larval dispersal used maps from cartopy [110], with the underlying vector map using NaturalEarth (naturalearthdata.com).
(TIF)

S23 Fig Particle trajectories. Example particle trajectories (*n* = 50) from Tenerife (Canary Islands, Spain) to the African shelf for dispersal durations of 102 (*i.e.*, dark, without benefits from the light-dependent activity of chromoplast components; left) and 181 (*i.e.*, light, with benefits from the light-dependent activity of chromoplast components; right) days. Grey arrows represent the 10-year mean (2007–2016) velocity for the release depth (0–30 m) from the VIKING20X model. Every 10th arrow is shown. Black contours mark the 500 m isobath around Africa. Visualizations of larval dispersal used maps from cartopy [110], with the underlying vector map using NaturalEarth (naturalearthdata.com).
(TIF)

S24 Fig Annual variation. Annual variation in particles reaching the African Shelf, as based on the VIKING20X model. The dashed gray line represents the mean for years 2007–2016 and the shaded areas mark one standard deviation. The vertical line indicates 102 days after release (*i.e.*, dark, without benefits from the light-dependent activity of chromoplast components).
(TIF)

S25 Fig Offspring spread more quickly. Modeled distribution of particles on the African shelf over multiple generations of dispersal. **(A)** Initial particles (*i.e.*, Generation 0) were released from Tenerife (Canary Islands, Spain), and were provided 102 (*i.e.*, dark, without benefits from the light-dependent activity of chromoplast components; left) or 181 (*i.e.*, light, with benefits from the light-dependent activity of chromoplast components; right) days to disperse. A second dispersal event of 102 or 181 days then initiated from their respective distributions. This was repeated until particles in each treatment reached the southernmost area of the range for the sea urchin *Arbacia lixula* (*i.e.*, the red area). **(B)** Offspring without benefits from the light-dependent activity of chromoplast components were close to reaching this on the Generation 4 (left) and did reach it on Generation 5, while those with benefits from the light-dependent activity of chromoplast components reached this area on Generation 3 **(C)**. Grey arrows represent the 10-year mean velocity for the release depth (0–30 m) from the VIKING20X. Every 10th arrow is shown. Black contours around the African shelf mark the 500 m isobath. Visualizations of larval dispersal used maps from cartopy [110], with the underlying vector map using NaturalEarth (naturalearthdata.com).
(TIF)

S26 Fig Zones of particle release off the coast of Africa. Particles were released from each of the four regions off the coast of Africa to determine if offspring with and/or without benefits from the light-dependent activity of chromoplast components could disperse across the Atlantic Ocean. Black contours mark the 500 m isobath around the African Coast. Visualizations of larval dispersal used maps from cartopy [110], with the underlying vector map using NaturalEarth (naturalearthdata.com).
(TIF)

S27 Fig Enabling trans-Atlantic dispersal. Distribution of particles in the Atlantic Ocean along the coast of Africa after 102 (*i.e.*, dark, without benefits from the light-dependent activity of chromoplast components; left) and 181 (*i.e.*, light, with benefits from the light-dependent activity of chromoplast components; right) days of being released. Grey arrows represent the 10-year mean velocity for the release depth (0–30 m) from VIKING20X. Every 50th arrow is shown. Black contours mark the 500 m isobath around Africa. Visualizations of larval dispersal used maps from cartopy [110], with the underlying vector map using NaturalEarth (naturalearthdata.com).
(TIF)

S28 Fig Annual variation. Annual variation in particles that disperse from the African Shelf to Brazil, as based on the VIKING20X model. The dashed gray line represents the mean for years 2007–2016 and the shaded areas mark one standard deviation. The vertical line indicates 102 days after release (*i.e.*, dark, without benefits from the light-dependent activity of chromoplast components).
(TIF)

S29 Fig Paths across the Atlantic Ocean. Distribution and abundance of particles that reach the coast of Brazil **(A)** and that depart from the coast of Africa **(B)** after 181 days of being released (*i.e.*, light, with benefits from the light-dependent activity of chromoplast components). This is a zoomed-in version of Fig 4B. Grey arrows represent the 10-year mean velocity for the release depth (0–30 m) from VIKING20X. Every 50th arrow is shown. Black contours mark the 500 m isobath around Africa. Visualizations of larval dispersal used maps from cartopy [110], with the underlying vector map using NaturalEarth (naturalearthdata.com).
(TIF)

S30 Fig Dispersal from Brazil. Distribution of particles in the Atlantic Ocean that were released along the northern coast of Brazil (red) after 181 (*i.e.*, light, with benefits from the light-dependent activity of chromoplast components) days of being released. Grey arrows represent the 10-year mean velocity for the release depth (0–30 m) from VIKING20X. Every 100th arrow is shown. Black contours represent 500 m isobath. Visualizations of larval dispersal used maps from cartopy [110], with the underlying vector map using NaturalEarth (naturalearthdata.com).
(TIF)

S31 Fig Unable to reach Macaronesia. Duration required for particles to disperse from the northern coast of Brazil to Macaronesia. A particle was first detected in Cape Verde on day 170, the Azores on day 366, and the Canary Islands on day 783. This is based on the VIKING20X model and is the accumulation of annual releases from years 2007 to 2016. Corresponding raw data are presented in S15 Table.
(TIF)

S32 Fig Rarefaction curves for amplicon sequencing. Rarefaction curves for the total ASVs **(A)** and phylogenetic diversity **(B)** of the microbial community associated with the developmental stages of the sea urchin *Arbacia lixula*. This was based on a rarefaction depth of 3,482 sequences and was used for all analyses. Corresponding raw data are presented in S16 Table.
(TIF)

S33 Fig Rarefaction curve for the metabolome. Rarefaction curve for the total metabolites observed based on the samples that were processed for the developmental stages of the sea urchin *Arbacia lixula*.
(TIF)

S1 Table Relative abundance of microbial groups in the developmental stages of *Arbacia lixula.* (XLSX)

S2 Table Meta-data for meta-analysis of phototrophic eukaryotes associated with sea urchin eggs. (XLSX)

S3 Table Relative abundance of Cyanobacteria and photosynthetic eukaryotes that are associated with sea urchin eggs. (XLSX)

S4 Table Relative abundance of plastid DNA in sea urchin eggs. This is from the meta-analysis.
(XLSX)

S5 Table List of plastid ASVs present in sea urchin eggs and their taxonomic assignment. This is from the meta-analysis.
(XLSX)

S6 Table Beta and alpha diversity comparisons between sea urchin eggs and the plastid ASVs that they are provided. This is from the meta-analysis.
(XLSX)

S7 Table Statistical tables for phylosymbiosis of plastid ASVs. This is from the meta-analysis.
(XLSX)

S8 Table Ribosomal counts (16S and 18S) from *Arbacia lixula* metagenome of embryos and larvae. Per offspring values were determined by dividing by 200.
(XLSX)

S9 Table List of metabolites detected in the eggs and larvae of *Arbacia lixula* from light and dark. (XLSX)

S10 Table Abundance of each metabolite detected in the eggs and larvae of *Arbacia lixula* from light and dark, as well as controls. (XLSX)

S11 Table Summary of statistical tests for sea urchin fitness (*i.e.*, development, morphology, and mortality). (XLSX)

S12 Table Summary of statistical tests for metabolomics. (XLSX)

S13 Table List of differentially abundance metabolites detected in the larvae of *Arbacia lixula* from light and dark. (XLSX)

S14 Table Setting for acquisition during LC-MS/MS metabolomics analysis. (XLSX)

S15 Table Dispersal quantification as part of an oceanographic model. (XLSX)

S16 Table Alpha rarefaction for the amplicon sequencing of *Arbacia lixula.* (XLSX)

## Abbreviations

ANOSIM: Analysis of Similarity
ASVs: amplicon sequence variants
COI: Cytochrome C Oxidase Subunit I
FSW: filtered seawater
LC–MS/MS: liquid chromatography tandem mass spectrometry
NCBI: National Center for Biotechnology Information
UHPLC: Ultra-High-Performance Liquid Chromatography
