# Supplementary figures and images for: Sea urchin eggs contain a plastid-derived structure that contributes to their development
Source: PLoS Biol. 2026 Apr 23;24(4):e3003705. doi: 10.1371/journal.pbio.3003705 (PMC13105349; doi:10.1371/journal.pbio.3003705)

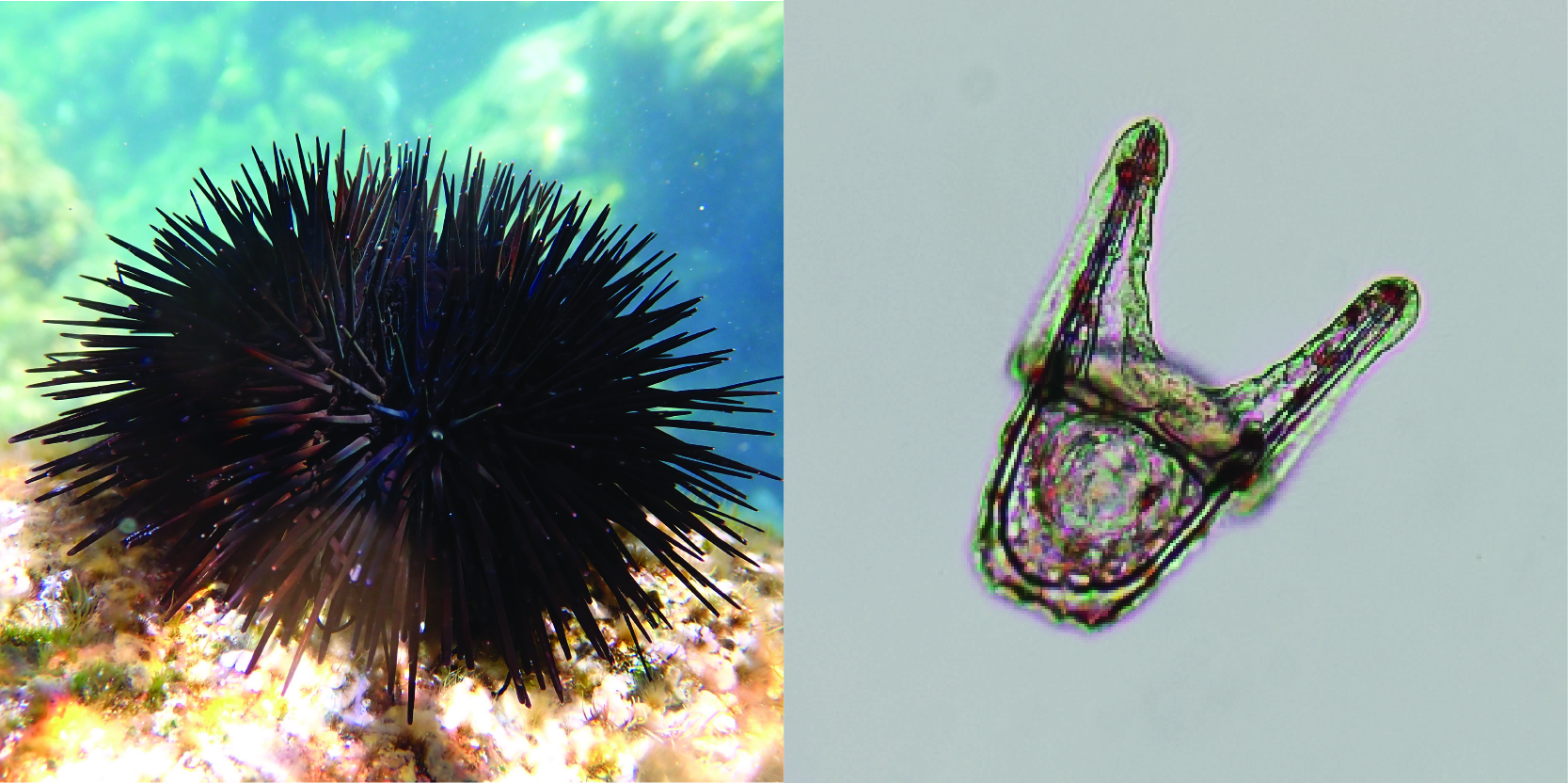

Supplement: S1 Fig — An adult (left) and larva (right) of the sea urchin Arbacia lixula. (TIF) [file pbio.3003705.s001.tif]

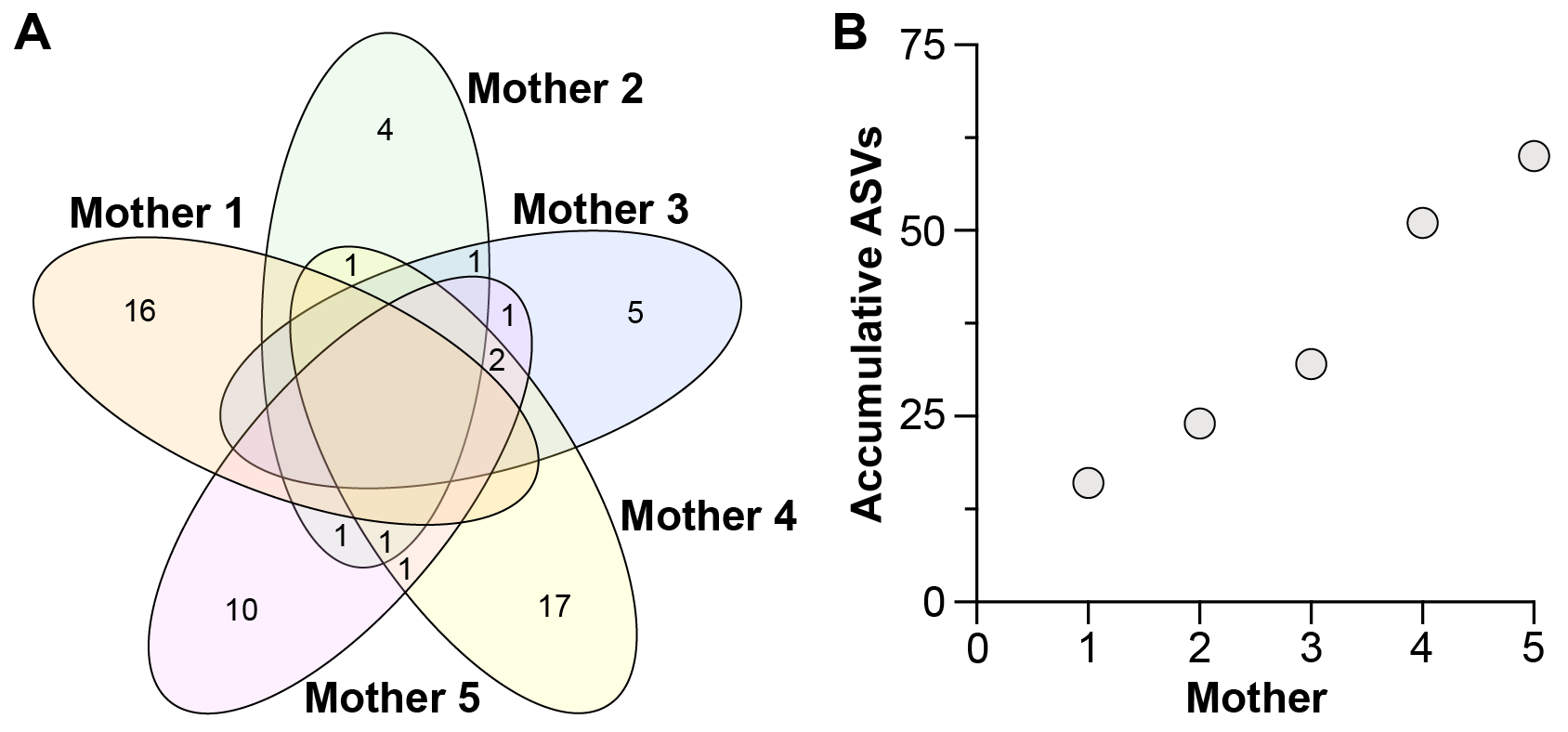

Supplement: S2 Fig — Minimal taxonomic overlap (A) and the diversity (B) of plastid ASVs in egg from different individuals of the sea urchin Arbacia lixula. Empty cells in the Venn diagram have zero ASVs. Corresponding raw data are presented in S1 Table. (TIF) [file pbio.3003705.s002.tif]

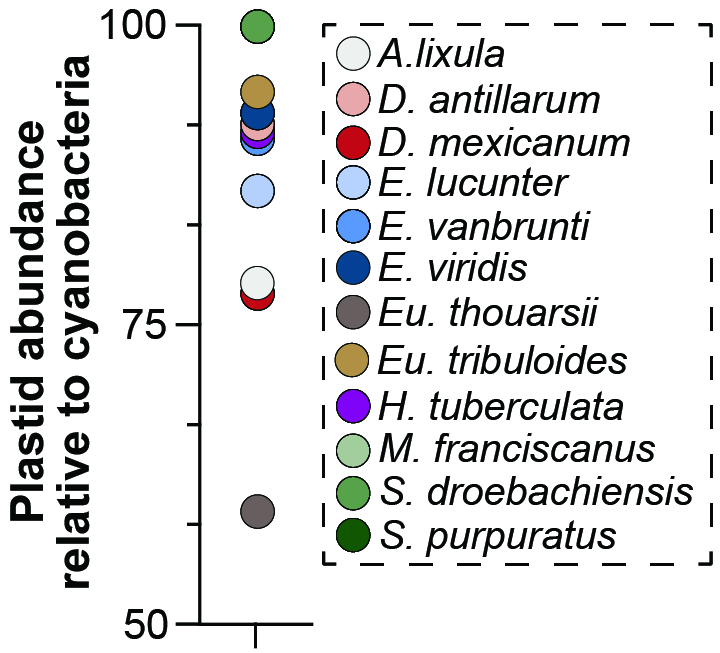

Supplement: S3 Fig — Abundance of plastid sequences from photosynthetic eukaryotes relative to that of cyanobacteria in the egg-associated microbiota of the 12 sea urchins that have been studied to date. Note: Heliocidaris erythrogramma that is present in S3 Fig was not included here because these taxonomic groups are not present. See S2 Table for replication. Corresponding raw data are presented in S3 Table. (TIF) [file pbio.3003705.s003.tif]

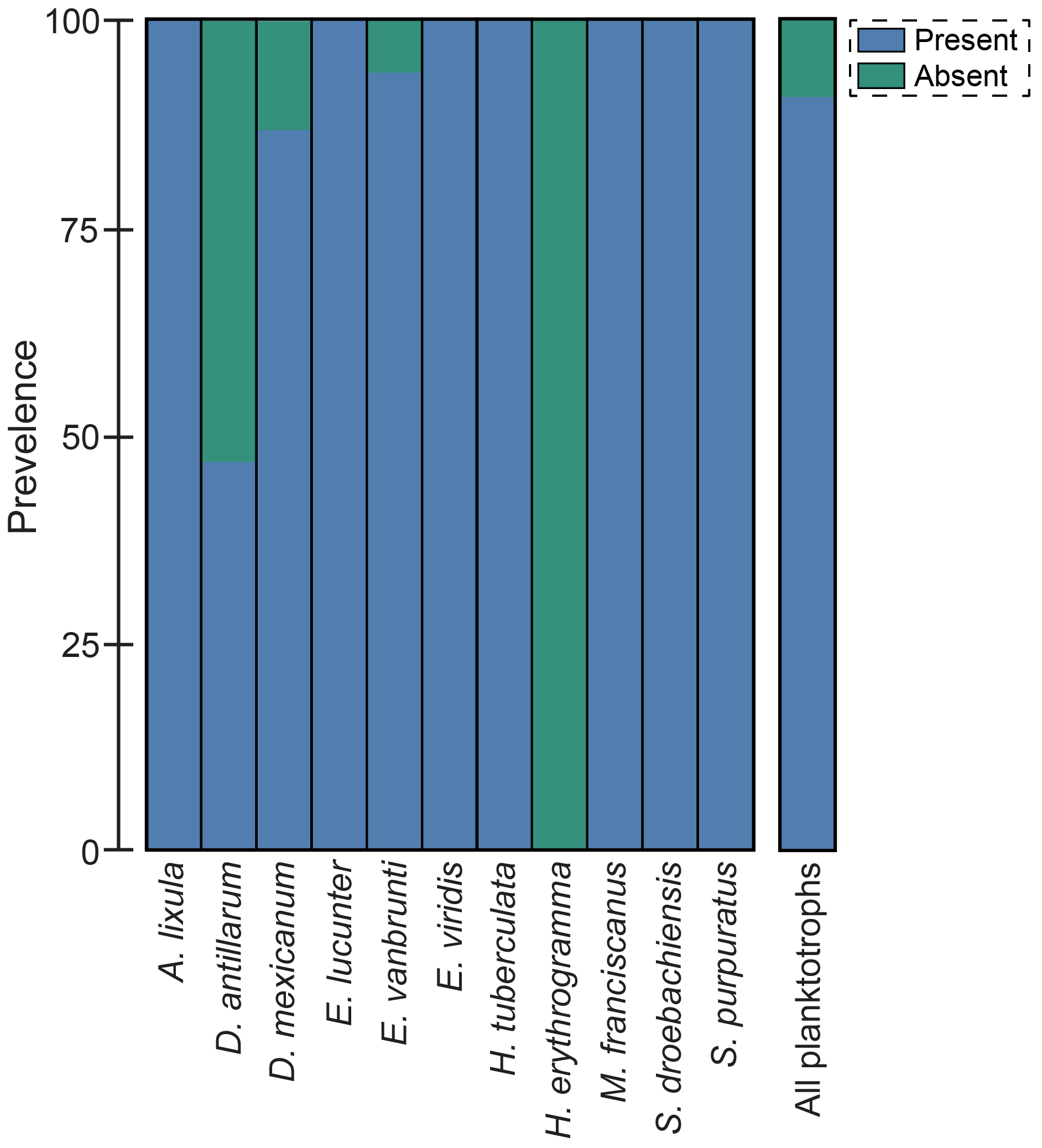

Supplement: S4 Fig — Plastid ASVs were present in the eggs of all but one sea urchin species, which was the only sea urchin that develops and undergoes metamorphosis without the requirement of external nutrients through feeding. The other species develop via feeding larvae, of which plastid DNA was present in 91% of these samples. Note: the two Eucidaris species in S2 Fig were not included in the rest of this meta-analysis because the single sample for each is insufficient for a full comparison. See S2 Table for replication and S3 Table for the corresponding raw data. (TIF) [file pbio.3003705.s004.tif]

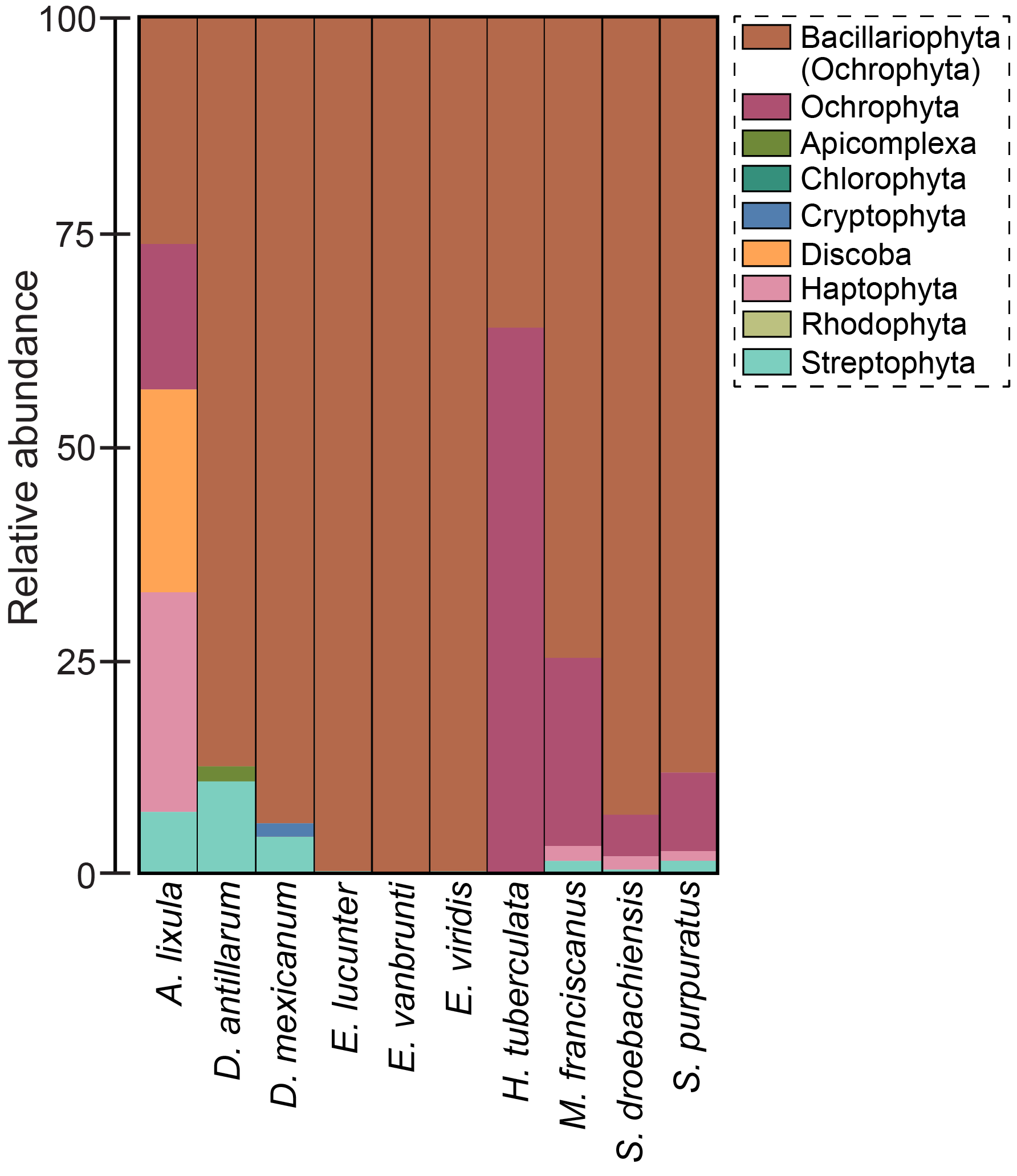

Supplement: S5 Fig — Taxonomic profile of the plastid ASVs from photosynthetic eukaryotes in sea urchin eggs. All groups represent eukaryotic phyla except the Bacillariophyta (diatoms), which are presented at the class-level and is part of the Ochrophtya. See S2 Table for replication and S4 Table for the corresponding raw data. (TIF) [file pbio.3003705.s005.tif]

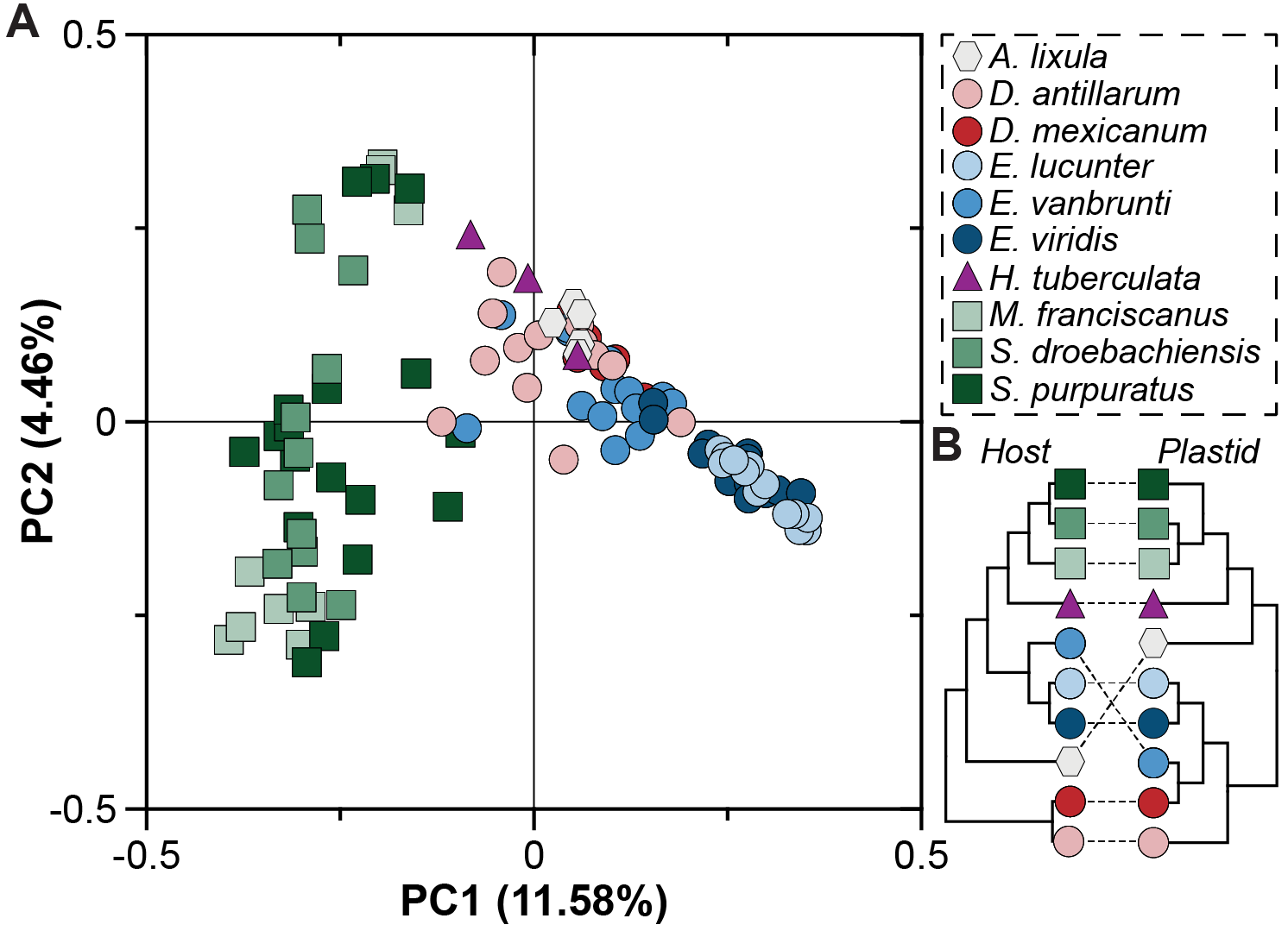

Supplement: S6 Fig — (A) Principal coordinate analysis depicting community relatedness (Jaccard) of the plastid ASVs in sea urchin eggs. Color and shape denote species and geographic locations, respectively. Square, circle, triangle, and hexagon represent Friday Harbor (WA, USA), Panama City and Colón (Panama), Sydney (Australia), and Tenerife (Canary Islands, Spain), respectively. (B) Topology of the host gene tree (using COI) is congruent with a dendrogram for these plastid ASVs based on species, even though they do not fully mirror each other. (TIF) [file pbio.3003705.s006.tif]

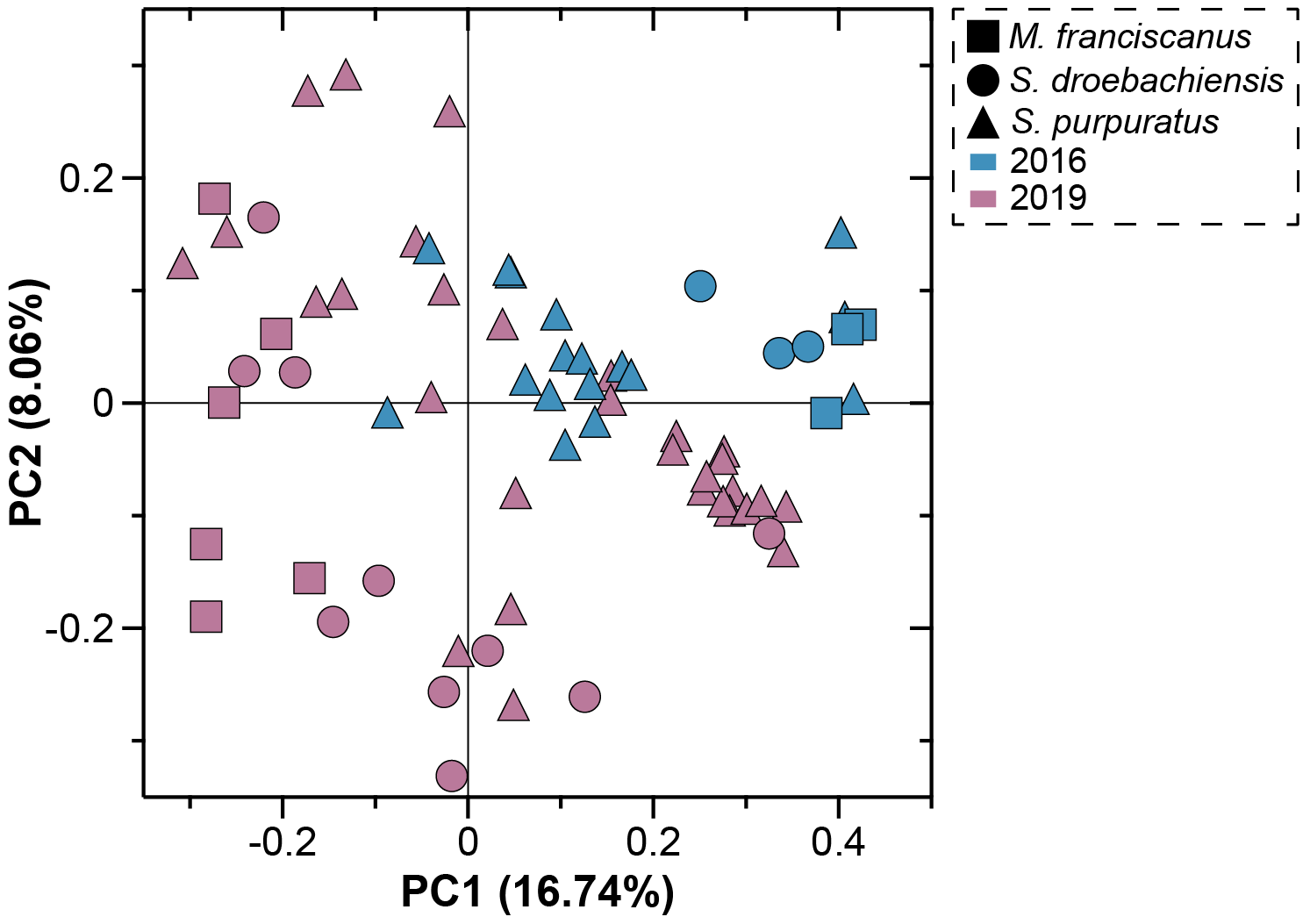

Supplement: S7 Fig — Principal coordinate analysis depicting community relatedness (Jaccard) of plastid ASVs that are associated with the eggs of the confamilials Strongylocentrotus purpuratus (triangle), Mesocentrotus franciscanus (square), and S. droebachiensis (circle) that were collected in 2016 (blue) and 2019 (pink) in Friday Harbor (WA, USA). (TIF) [file pbio.3003705.s007.tif]

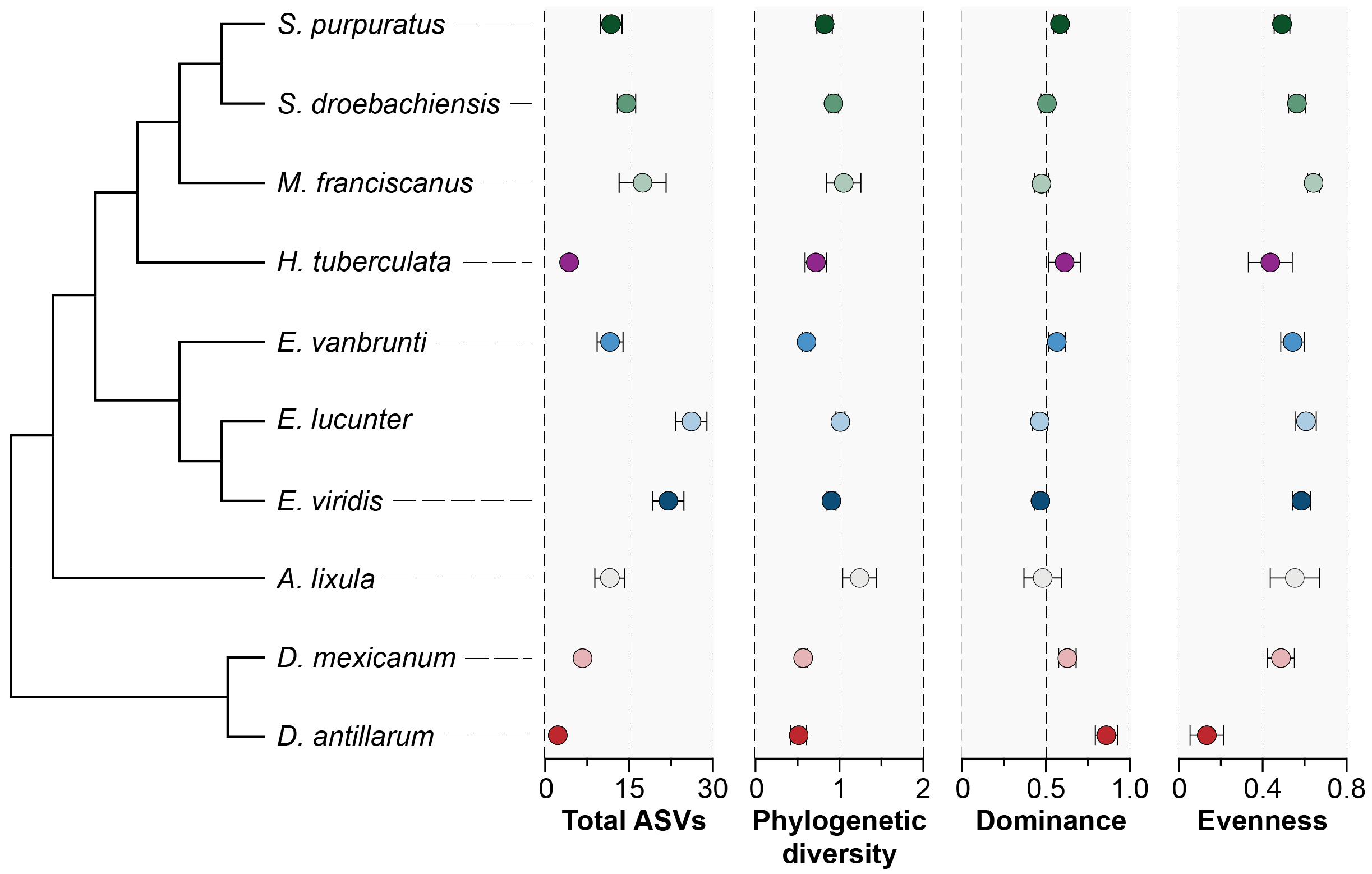

Supplement: S8 Fig — This was estimated by total ASVs, Faith’s phylogenetic diversity, McIntosh dominance, and McIntosh evenness. Each circle species average ± standard error. Corresponding raw data are presented in S6 Table. (TIF) [file pbio.3003705.s008.tif]

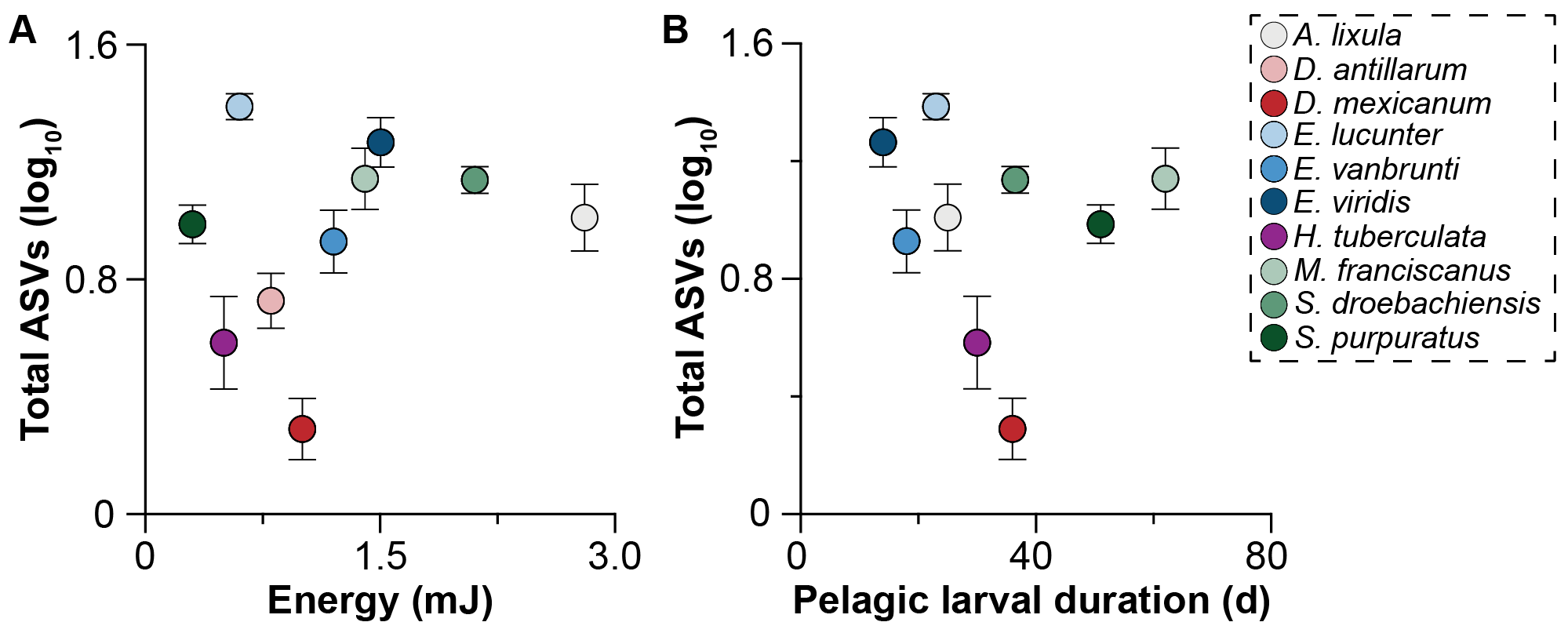

Supplement: S9 Fig — No trade-off was observed between the energetic content (C; mJ; linear regression: F1,109 = 1.36, p = 0.246, R2 = 0.012) of sea urchin eggs or pelagic larval duration (D; days; linear regression: F1,109 = 0.380, p = 0.539, R2 = 0.003) and the diversity of plastid ASVs that were provided to sea urchin eggs. Each circle represent a species average with their corresponding standard error bar. Corresponding raw data are presented in S6 Table. (TIF) [file pbio.3003705.s009.tif]

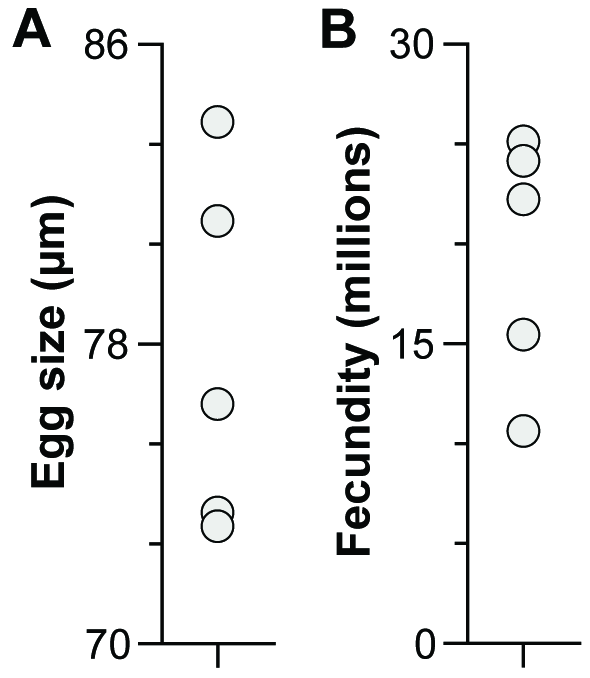

Supplement: S10 Fig — Average egg size (diameter; µm) (A) and fecundity (B) for five individuals of the sea urchin Arbacia lixula. Corresponding raw data are presented in S11 Table. (TIF) [file pbio.3003705.s010.tif]

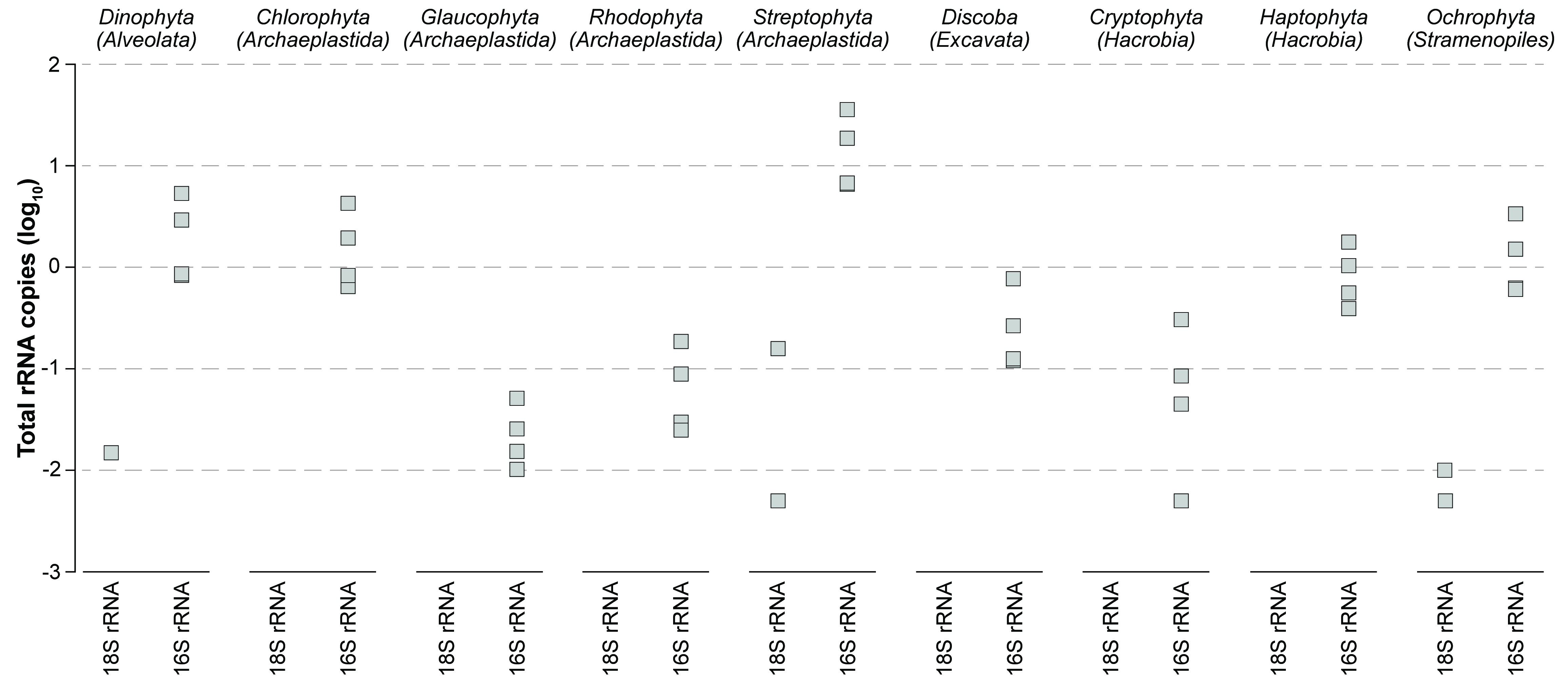

Supplement: S11 Fig — A nuclear gene marker (18S rRNA gene) was disproportionately low in abundance or not present in embryos of the sea urchin Arbacia lixula compared to a plastid gene marker (16S rRNA gene) for the Alveolata (Dinophyta), Archaeplastida (Chlorophyta, Glaucophyta, Rhodophyta, Streptophyta), Excavata (Discoba), Hacrobia (Cryptophyta, Haptophyta), and Stramenopiles (Ochrophyta). This is a group-by-group display of the rRNA counts presented in Fig 1C, with corresponding raw data being presented in S8 Table. (TIF) [file pbio.3003705.s011.tif]

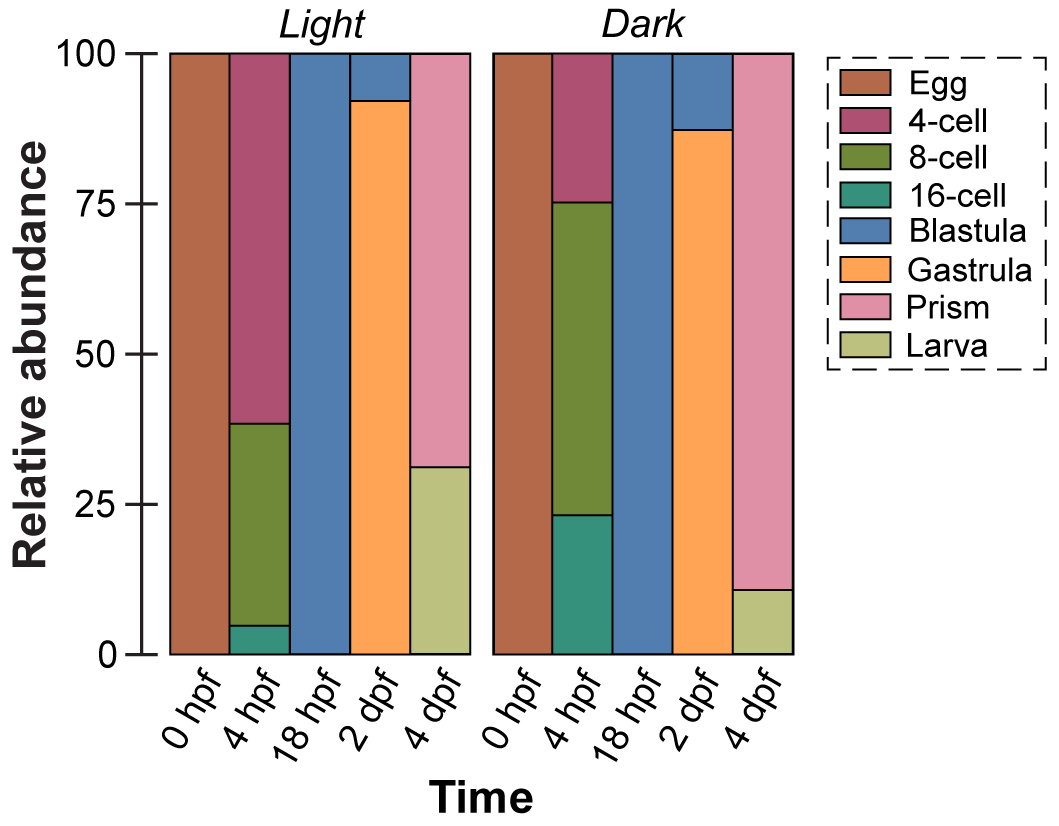

Supplement: S14 Fig — The developmental stages for offspring of the sea urchin Arbacia lixula were recorded at several intervals while being cultured from fertilization through four days post-fertilization in light (i.e., with benefits from the light-dependent activity of chromoplast-derived components) and dark (i.e., without benefits from the light-dependent activity of chromoplast-derived components). Corresponding raw data are presented in S11 Table. (TIF) [file pbio.3003705.s014.tif]

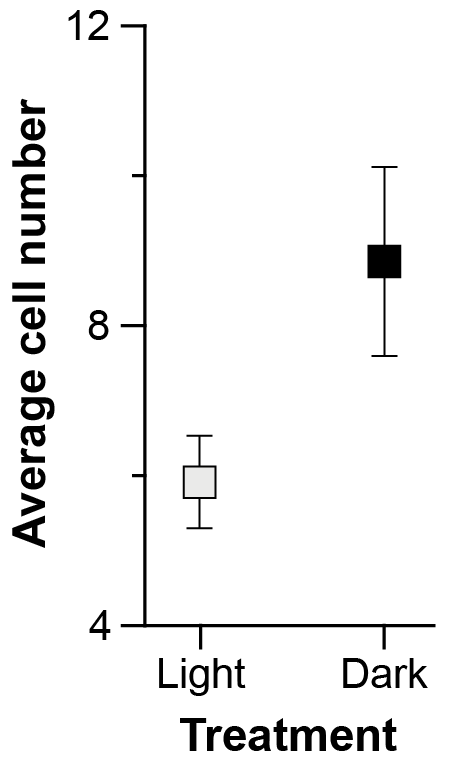

Supplement: S15 Fig — Offspring develop quicker in dark (i.e., without benefits from the light-dependent activity of chromoplast-derived components) during the first four cell divisions (i.e., from fertilization to the 16-cell stage), as compared to their siblings in light (i.e., with benefits from the light-dependent activity of chromoplast-derived components). All values are average ± standard error. These data are from 4 hours post-fertilization (S14 Fig). Corresponding raw data are presented in S11 Table. (TIF) [file pbio.3003705.s015.tif]

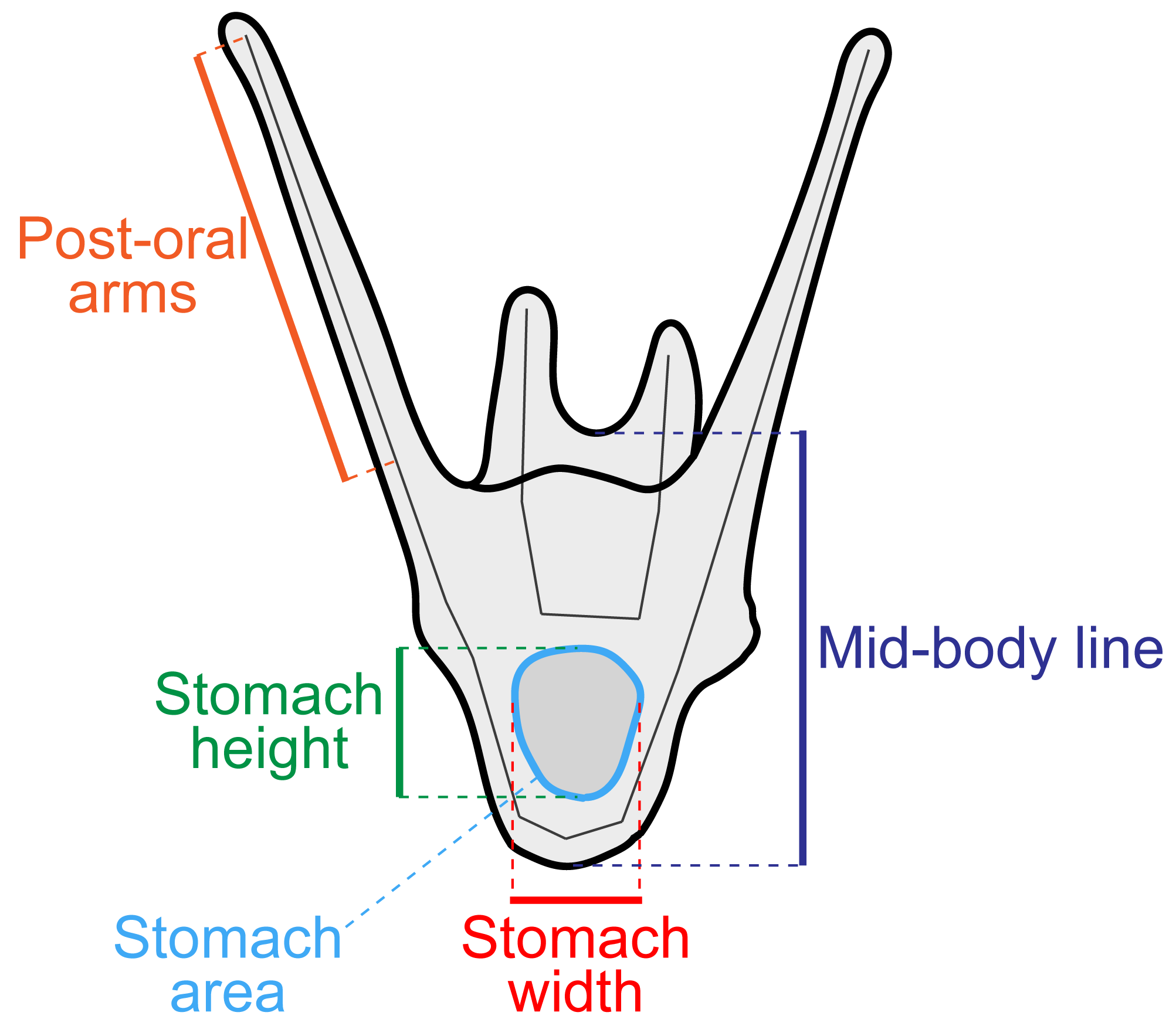

Supplement: S16 Fig — Schematic of the morphological features that were measured to assess whether the two-arm larvae (four days post-fertilization) of the sea urchin Arbacia lixula exhibited morphological plasticity. (TIF) [file pbio.3003705.s016.tif]

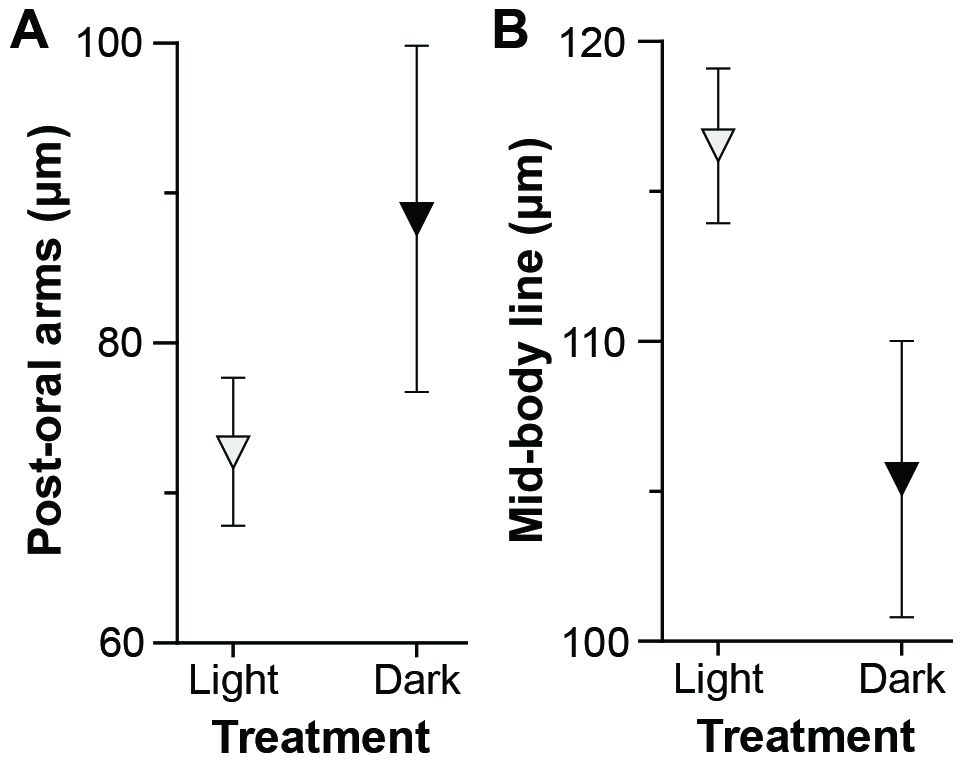

Supplement: S17 Fig — The length of the post-oral arms (A) and the mid-body line (B) were similar between treatments for larvae of the sea urchin Arbacia lixula that were cultured in light (i.e., with benefits from the light-dependent activity of chromoplast-derived components), as compared to their siblings in dark (i.e., without benefits from the light-dependent activity of chromoplast-derived components). All values are average ± standard error (i.e., the combination across all larvae from light or dark replicates; n = 49 in light and n = 10 in dark). Corresponding raw data are presented in S11 Table. (TIF) [file pbio.3003705.s017.tif]

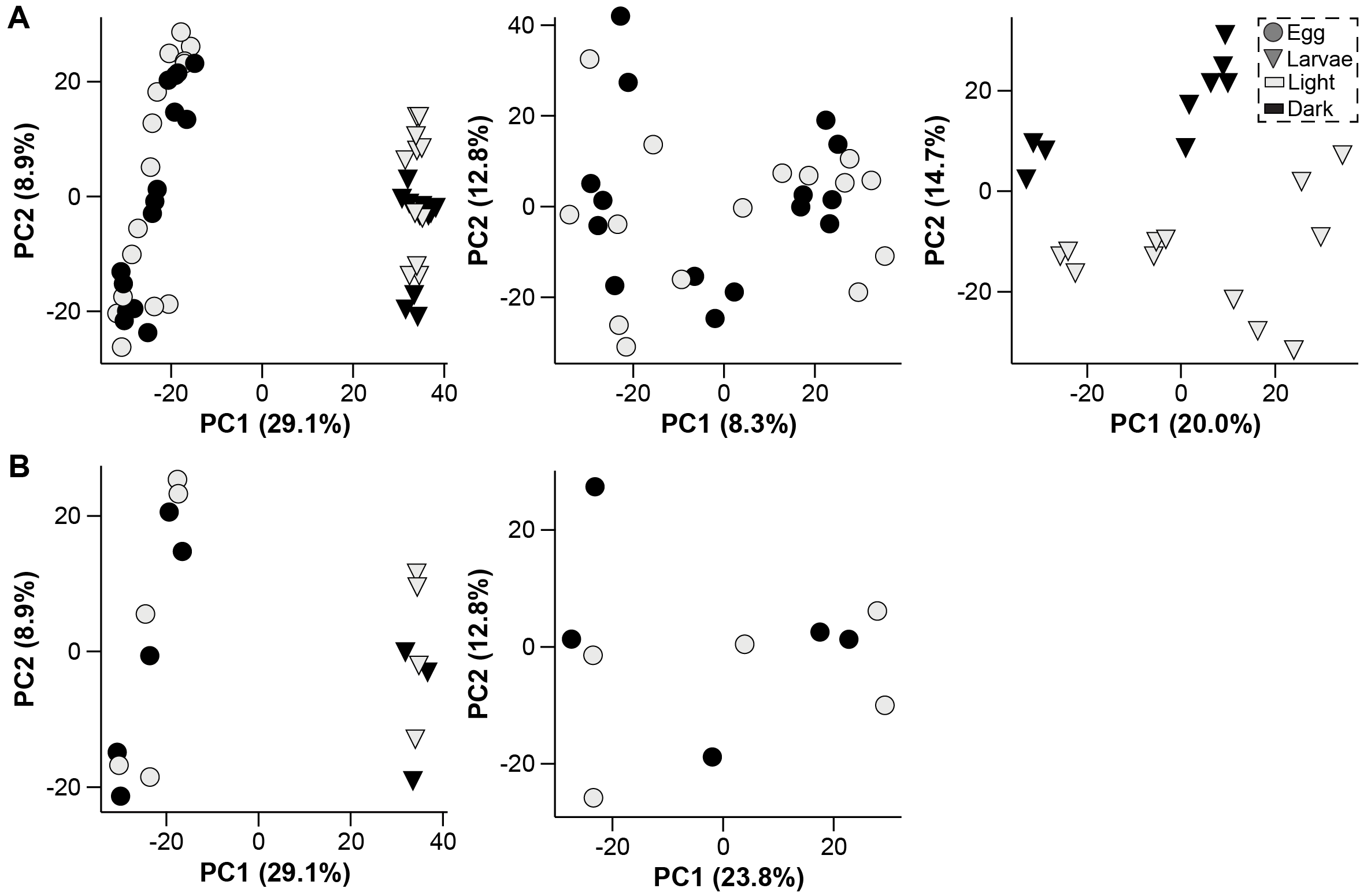

Supplement: S18 Fig — Eggs of the sea urchin Arbacia lixula that were placed in light (i.e., with benefits from the light-dependent activity of chromoplast-derived components) and dark (i.e., without benefits from the light-dependent activity of chromoplast-derived components) had similar metabolomes (left and center). A metabolome-wide shift occurred during development (left) and based on the presence of light for larvae (right). These PCA plots are displayed with each technical replicate (A) as well as the median of those technical replicates (B). Larvae with the median of their technical replicates is Fig 3D. (TIF) [file pbio.3003705.s018.tif]

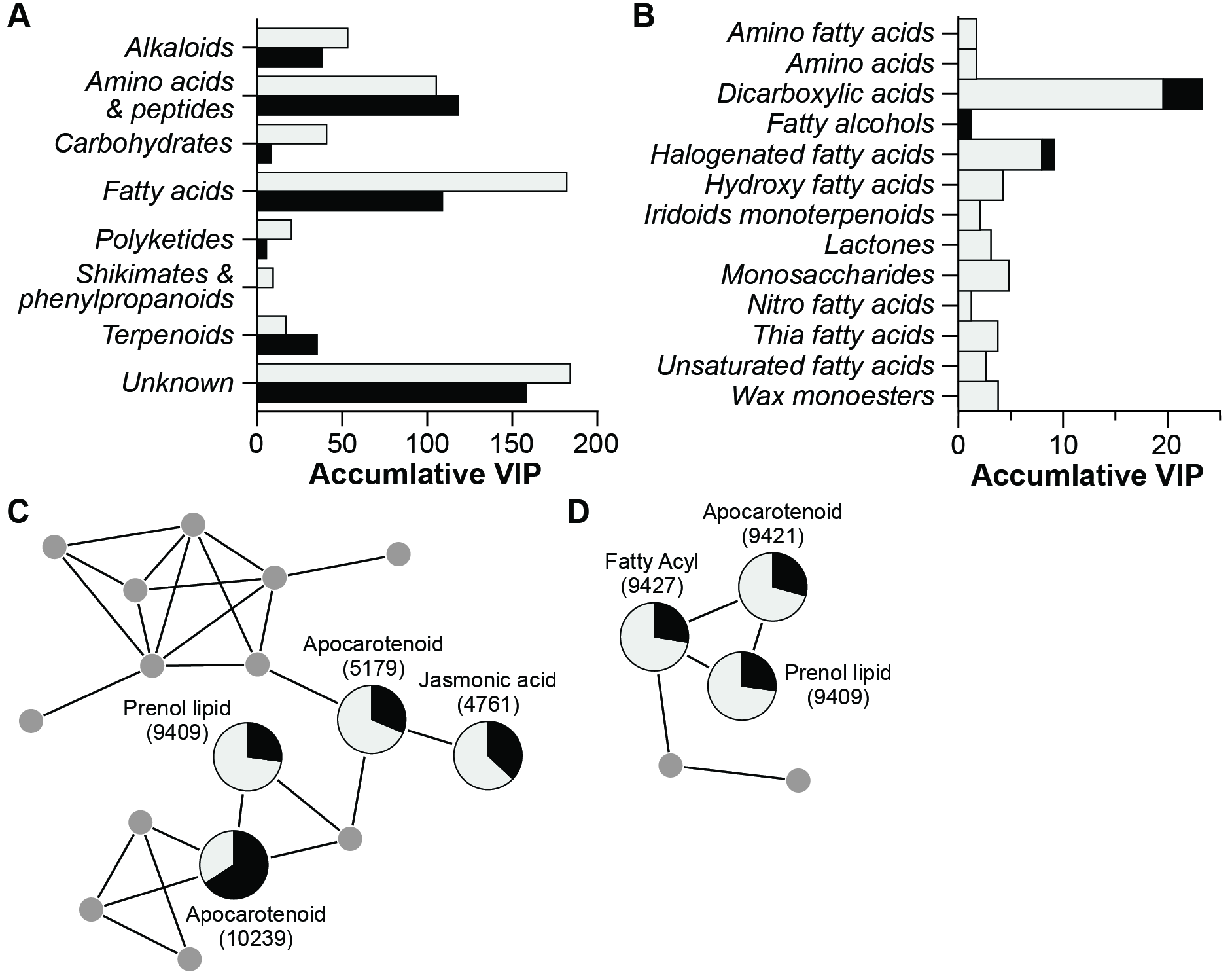

Supplement: S19 Fig — (A) Larvae cultured in light and dark exhibit organism-wide differences in their metabolism. (B) This was predominantly driven by a shift in fatty acid metabolism, including dicarboxylic acids as well as several other complementary fatty acids that were mainly differentially abundant in light. (C) Metabolite 9409 (a prenol lipid) was part of a metabolic module that had three other known metabolites that were found in the offspring of the sea urchin Arbacia lixula. This module included two uncharacterized apocarotenoids and jasmonic acid, all of which are phytohormones. (D) Metabolite 9421 was part of a metabolic module that included a prenol lipid and fatty acyl. Corresponding raw data are presented in S9, S10, and S13 Tables. (TIF) [file pbio.3003705.s019.tif]

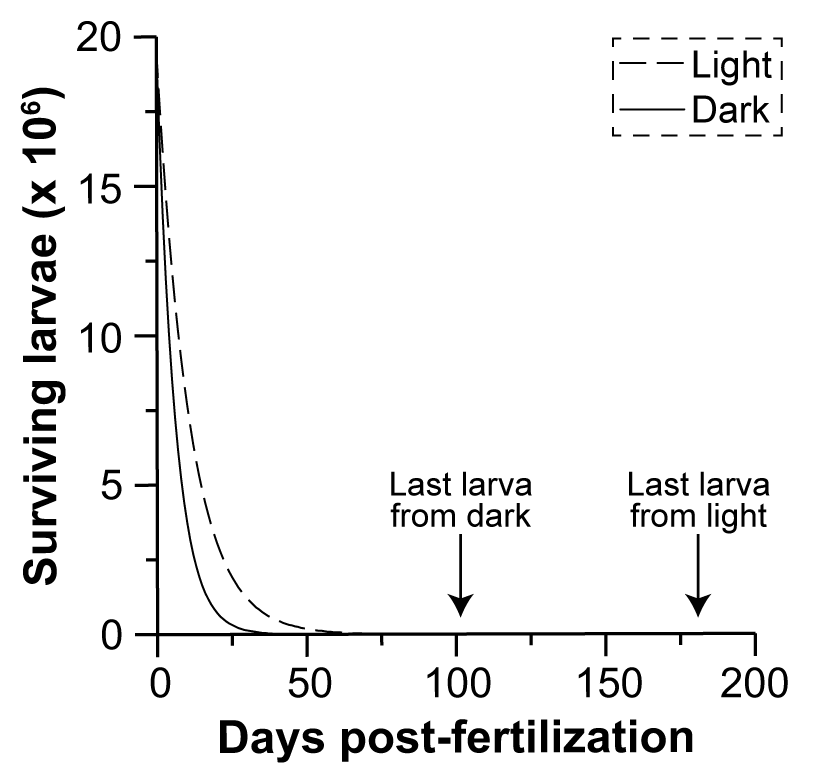

Supplement: S20 Fig — Planktonic larval duration was estimated for development in light (i.e., with benefits from the light-dependent activity of chromoplast components) and dark (i.e., without benefits from the light-dependent activity of chromoplast components) using the fecundity of the sea urchin Arbacia lixula and the instantaneous rate model for the natural mortality of marine invertebrate larvae. It is estimated to take 181 and 102 days in light and dark, respectively, for the last larva from an entire clutch of an individual A. lixula to remain. Visualizations of larval dispersal used maps from cartopy [110], with the underlying vector map using NaturalEarth (naturalearthdata.com). Corresponding raw data are presented in S11 Table. (TIF) [file pbio.3003705.s020.tif]

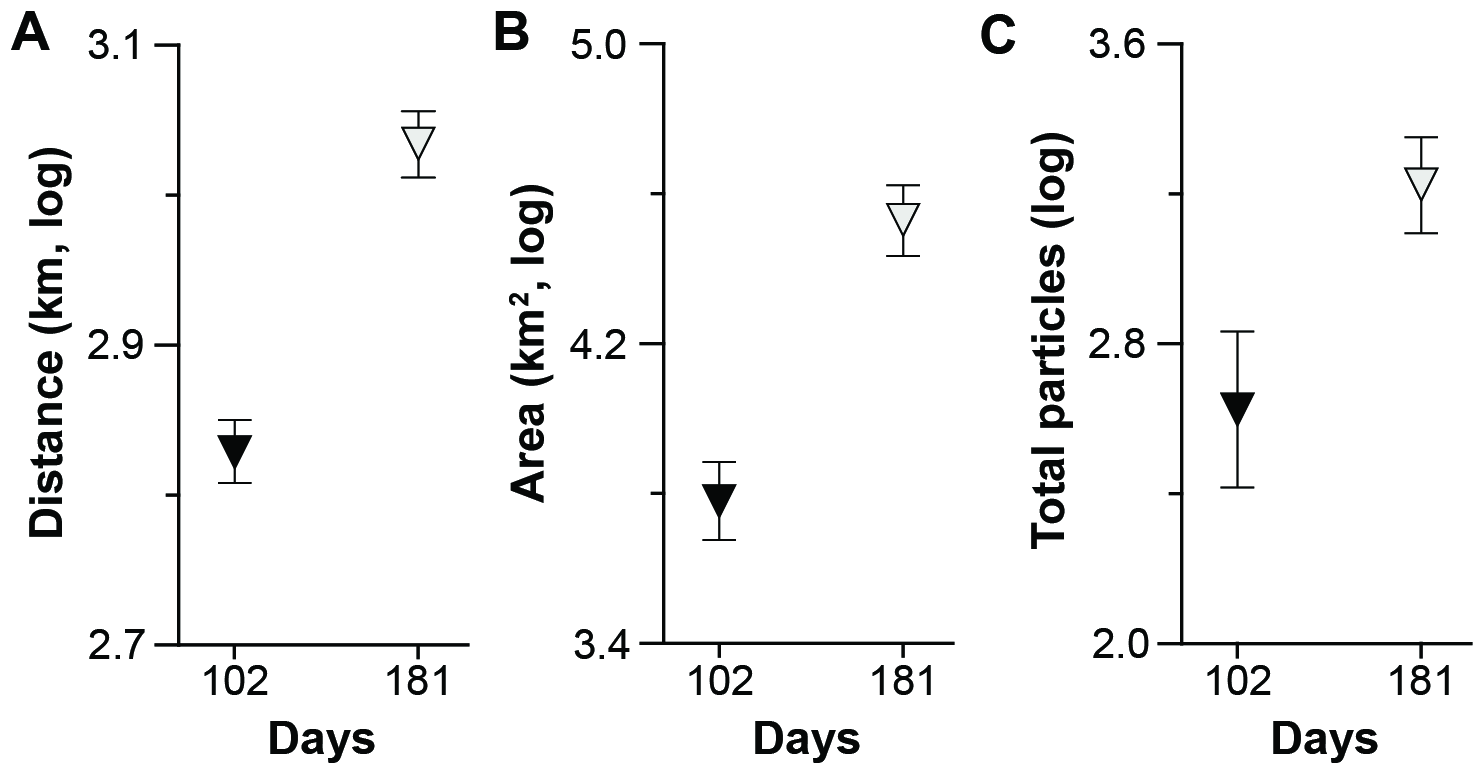

Supplement: S21 Fig — Offspring cultured in light (i.e., with benefits from the light-dependent activity of chromoplast components) enhances the total dispersal distance (A), geographical area that is settled on (B), and abundance of particles reaching a habitat to settle (C), as compared to their siblings in dark (i.e., without benefits from the light-dependent activity of chromoplast components). These numerical values were extracted from Fig 4A, of which are derived from the distribution of particles on the African shelf after 102 (i.e., without benefits from the light-dependent activity of chromoplast components) and 181 (i.e., with benefits from the light-dependent activity of chromoplast components) days of being released from Tenerife (Canary Islands, Spain). All data were log-transformed for normality. Each dot represents the 10-year average ± standard error. Visualizations of larval dispersal used maps from cartopy [110], with the underlying vector map using NaturalEarth (naturalearthdata.com). Corresponding raw data are presented in S15 Table. (TIF) [file pbio.3003705.s021.tif]

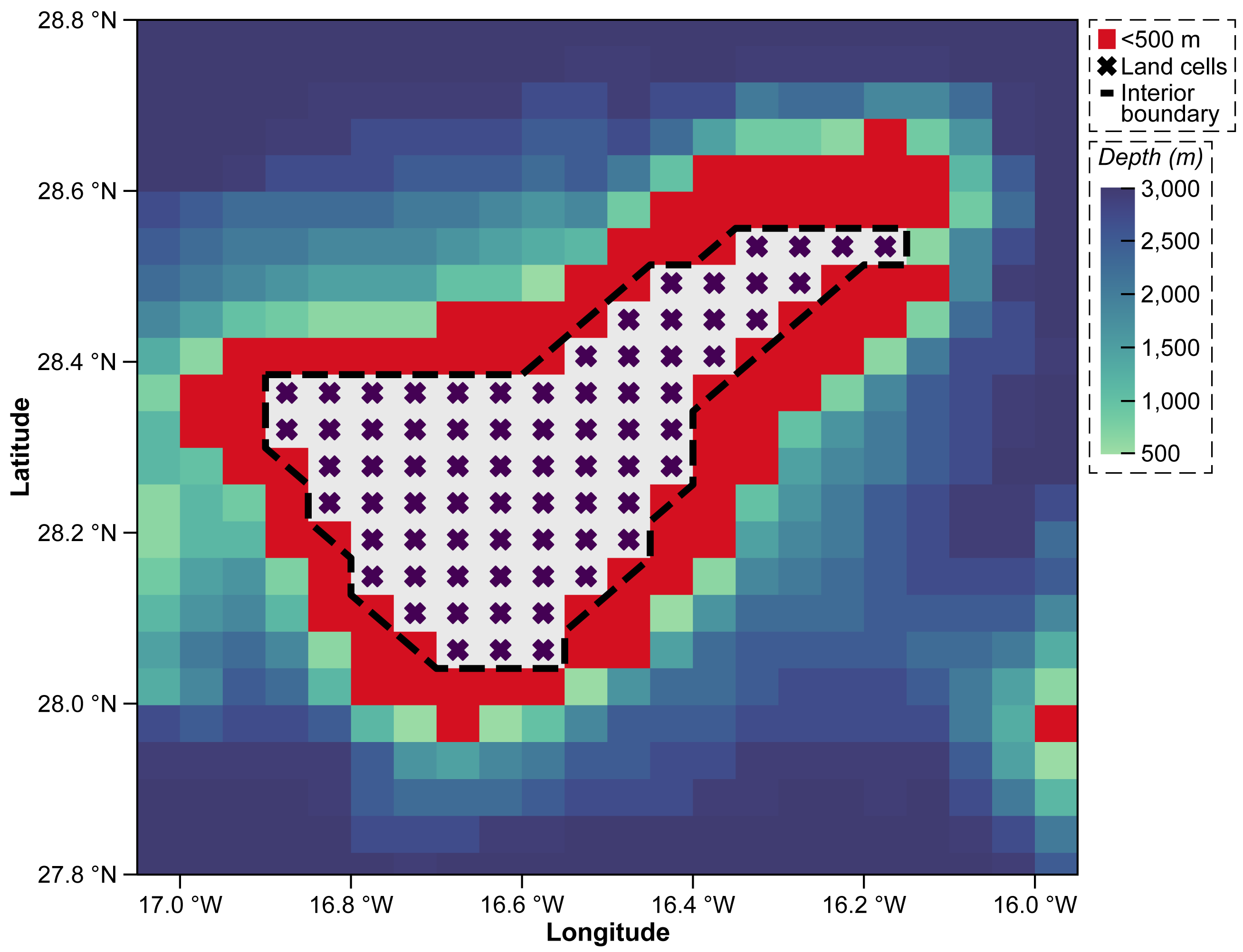

Supplement: S22 Fig — Location of particle release from Tenerife (Canary Islands, Spain) at the grid resolution of the VIKING20X model. Particles were released from grid cells that were <500 m (red squares) adjacent to Tenerife. Visualizations of larval dispersal used maps from cartopy [110], with the underlying vector map using NaturalEarth (naturalearthdata.com). (TIF) [file pbio.3003705.s022.tif]

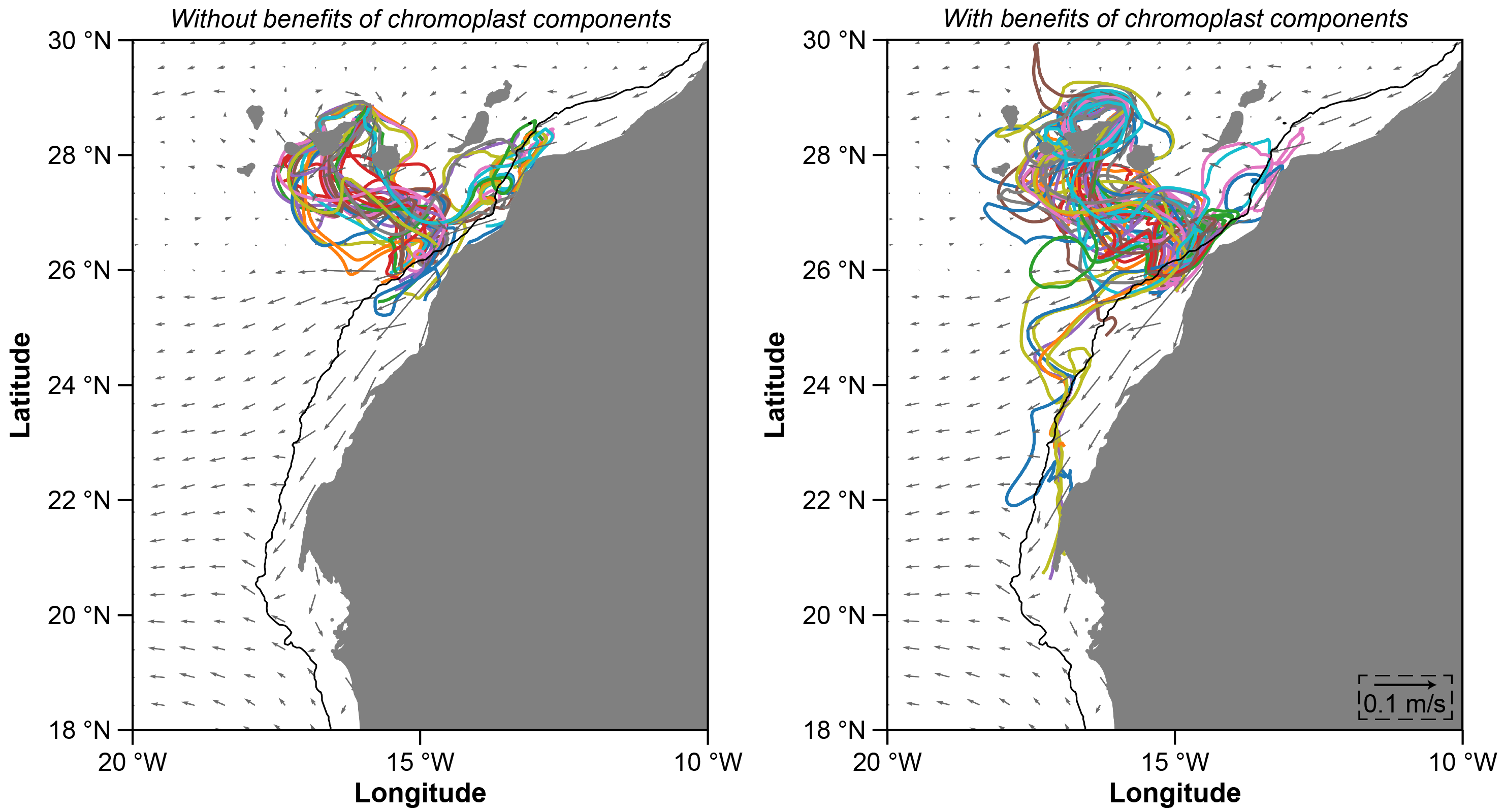

Supplement: S23 Fig — Example particle trajectories (n = 50) from Tenerife (Canary Islands, Spain) to the African shelf for dispersal durations of 102 (i.e., dark, without benefits from the light-dependent activity of chromoplast components; left) and 181 (i.e., light, with benefits from the light-dependent activity of chromoplast components; right) days. Grey arrows represent the 10-year mean (2007–2016) velocity for the release depth (0–30 m) from the VIKING20X model. Every 10th arrow is shown. Black contours mark the 500 m isobath around Africa. Visualizations of larval dispersal used maps from cartopy [110], with the underlying vector map using NaturalEarth (naturalearthdata.com). (TIF) [file pbio.3003705.s023.tif]

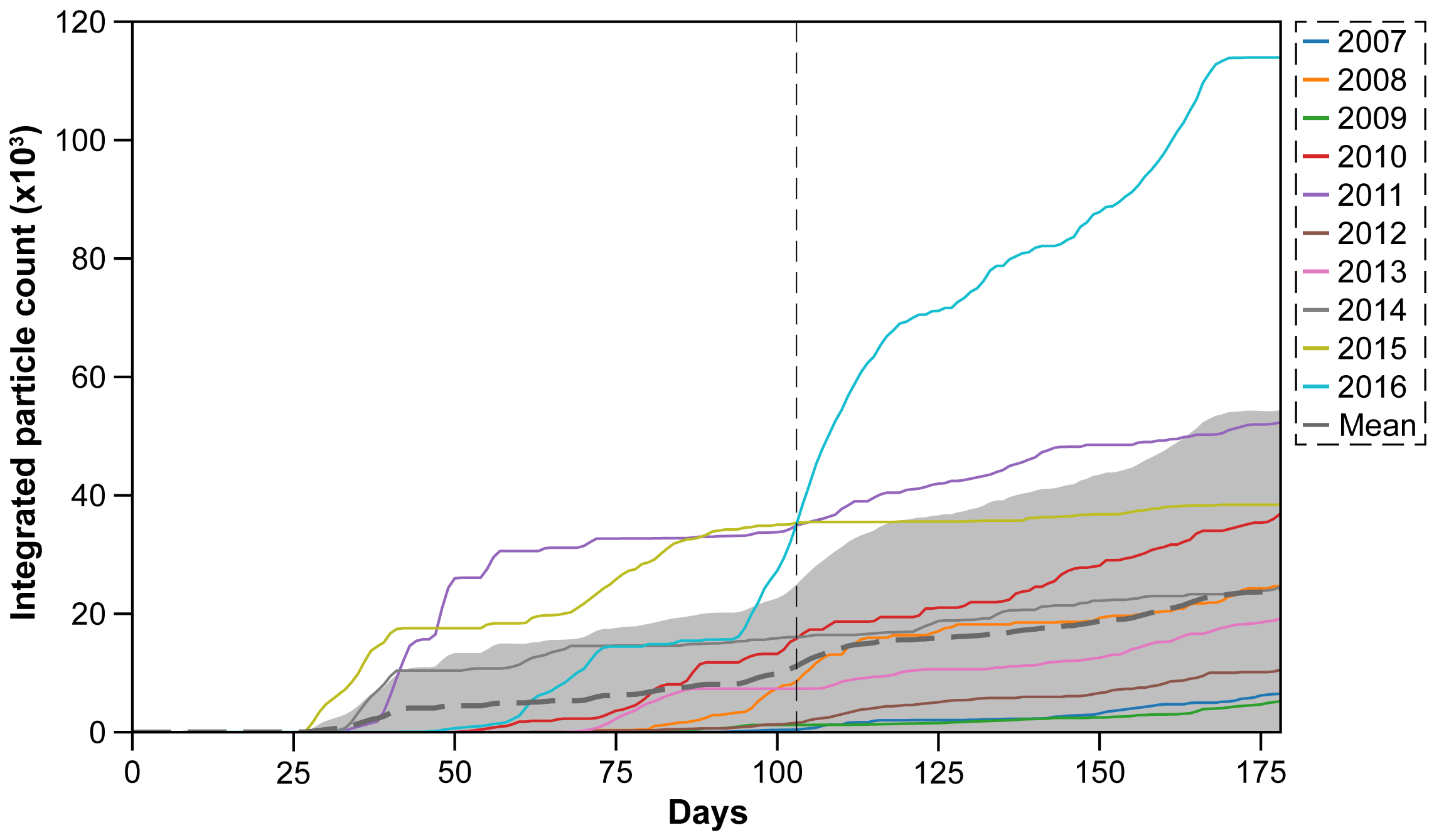

Supplement: S24 Fig — Annual variation in particles reaching the African Shelf, as based on the VIKING20X model. The dashed gray line represents the mean for years 2007–2016 and the shaded areas mark one standard deviation. The vertical line indicates 102 days after release (i.e., dark, without benefits from the light-dependent activity of chromoplast components). (TIF) [file pbio.3003705.s024.tif]

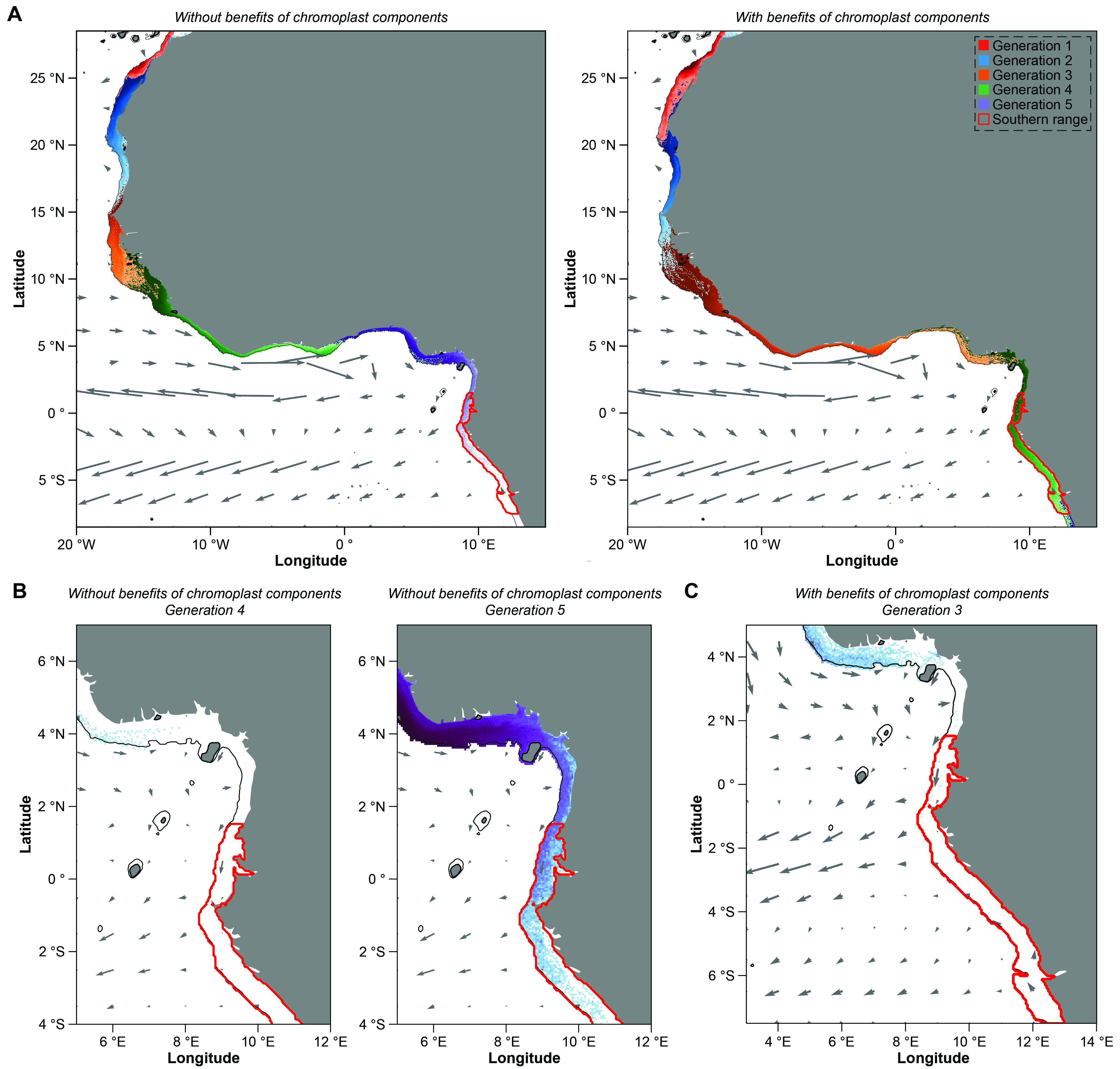

Supplement: S25 Fig — Modeled distribution of particles on the African shelf over multiple generations of dispersal. (A) Initial particles (i.e., Generation 0) were released from Tenerife (Canary Islands, Spain), and were provided 102 (i.e., dark, without benefits from the light-dependent activity of chromoplast components; left) or 181 (i.e., light, with benefits from the light-dependent activity of chromoplast components; right) days to disperse. A second dispersal event of 102 or 181 days then initiated from their respective distributions. This was repeated until particles in each treatment reached the southernmost area of the range for the sea urchin Arbacia lixula (i.e., the red area). (B) Offspring without benefits from the light-dependent activity of chromoplast components were close to reaching this on the Generation 4 (left) and did reach it on Generation 5, while those with benefits from the light-dependent activity of chromoplast components reached this area on Generation 3 (C). Grey arrows represent the 10-year mean velocity for the release depth (0–30 m) from the VIKING20X. Every 10th arrow is shown. Black contours around the African shelf mark the 500 m isobath. Visualizations of larval dispersal used maps from cartopy [110], with the underlying vector map using NaturalEarth (naturalearthdata.com). (TIF) [file pbio.3003705.s025.tif]

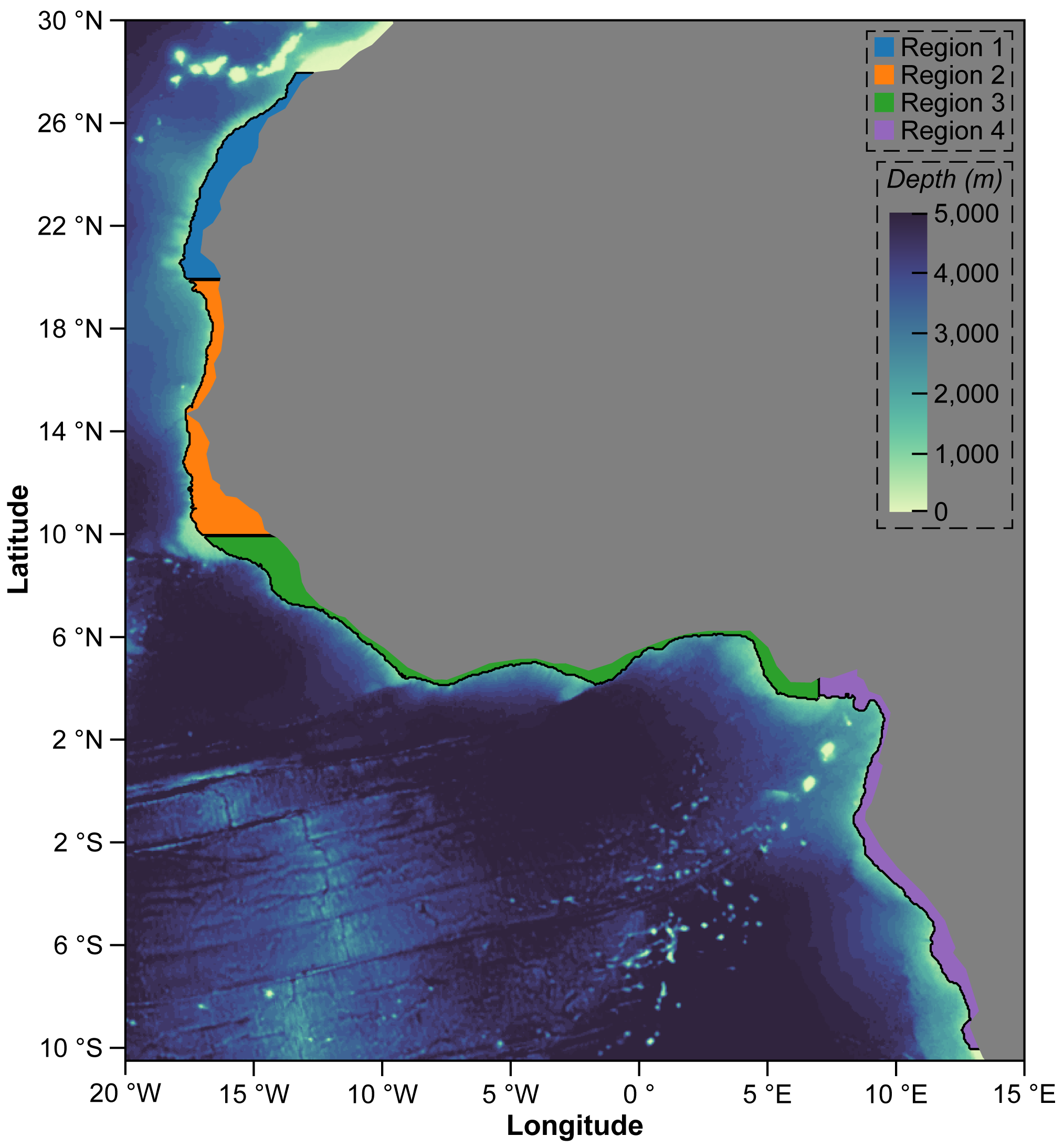

Supplement: S26 Fig — Particles were released from each of the four regions off the coast of Africa to determine if offspring with and/or without benefits from the light-dependent activity of chromoplast components could disperse across the Atlantic Ocean. Black contours mark the 500 m isobath around the African Coast. Visualizations of larval dispersal used maps from cartopy [110], with the underlying vector map using NaturalEarth (naturalearthdata.com). (TIF) [file pbio.3003705.s026.tif]

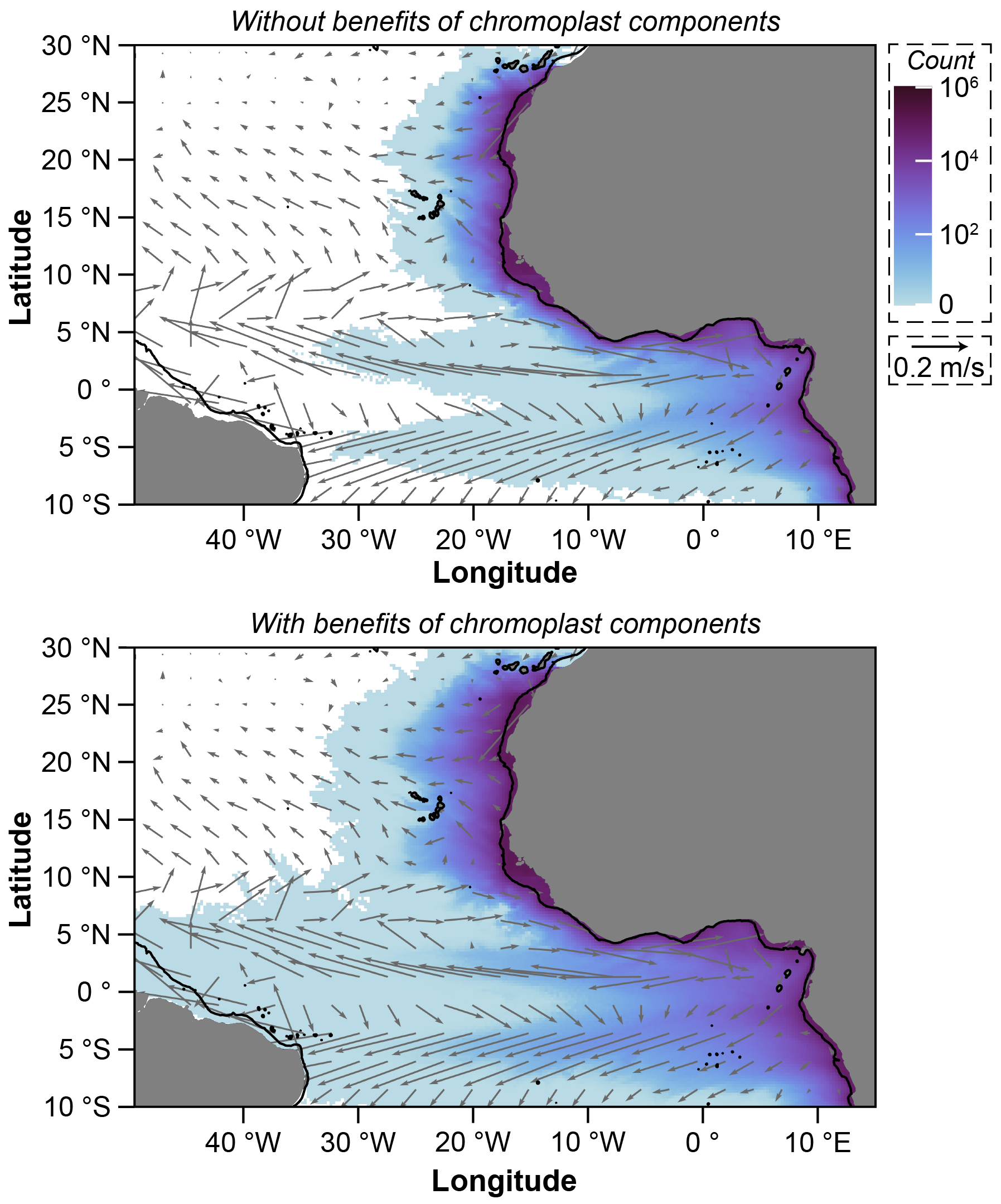

Supplement: S27 Fig — Distribution of particles in the Atlantic Ocean along the coast of Africa after 102 (i.e., dark, without benefits from the light-dependent activity of chromoplast components; left) and 181 (i.e., light, with benefits from the light-dependent activity of chromoplast components; right) days of being released. Grey arrows represent the 10-year mean velocity for the release depth (0–30 m) from VIKING20X. Every 50th arrow is shown. Black contours mark the 500 m isobath around Africa. Visualizations of larval dispersal used maps from cartopy [110], with the underlying vector map using NaturalEarth (naturalearthdata.com). (TIF) [file pbio.3003705.s027.tif]

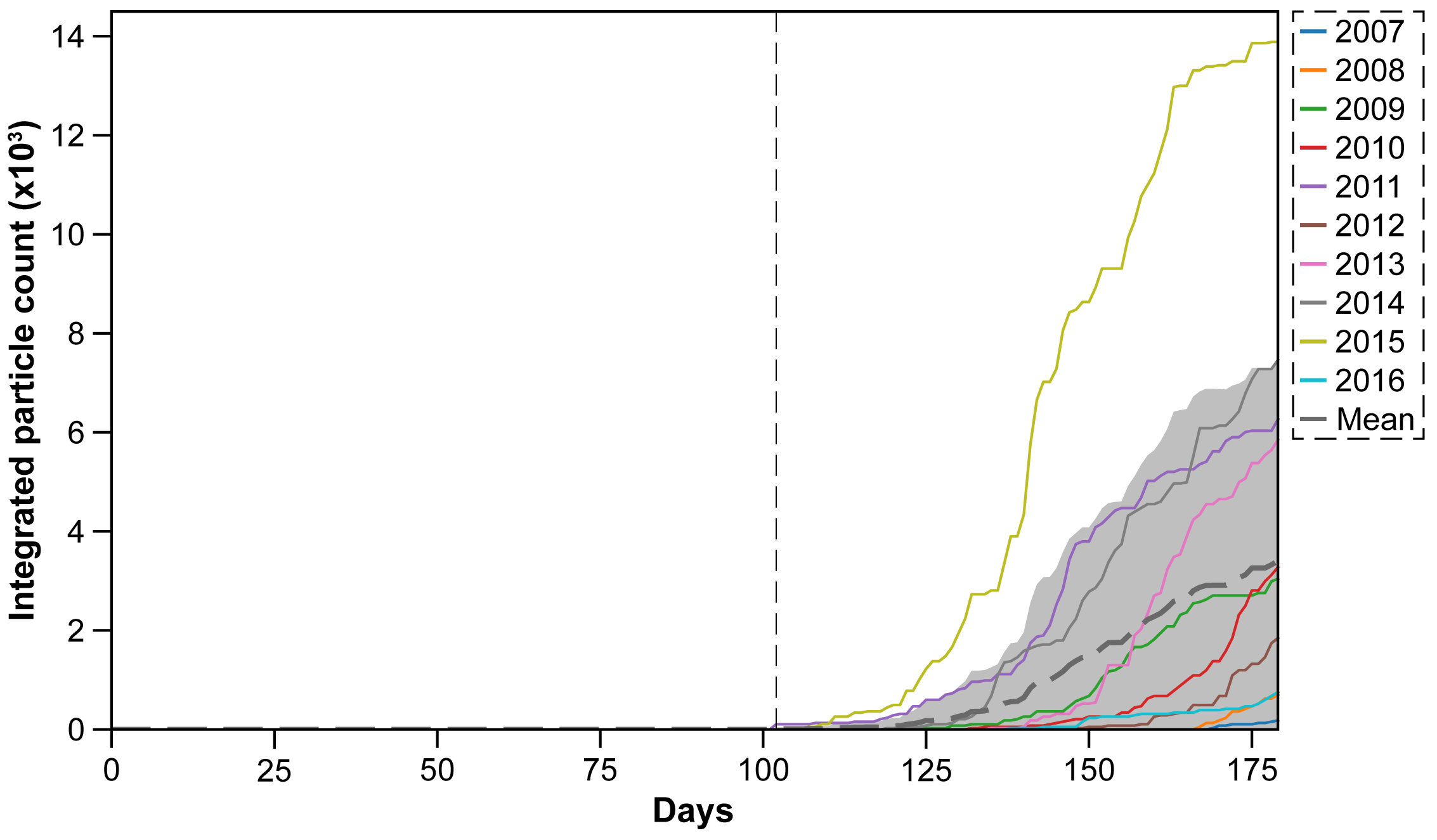

Supplement: S28 Fig — Annual variation in particles that disperse from the African Shelf to Brazil, as based on the VIKING20X model. The dashed gray line represents the mean for years 2007–2016 and the shaded areas mark one standard deviation. The vertical line indicates 102 days after release (i.e., dark, without benefits from the light-dependent activity of chromoplast components). (TIF) [file pbio.3003705.s028.tif]

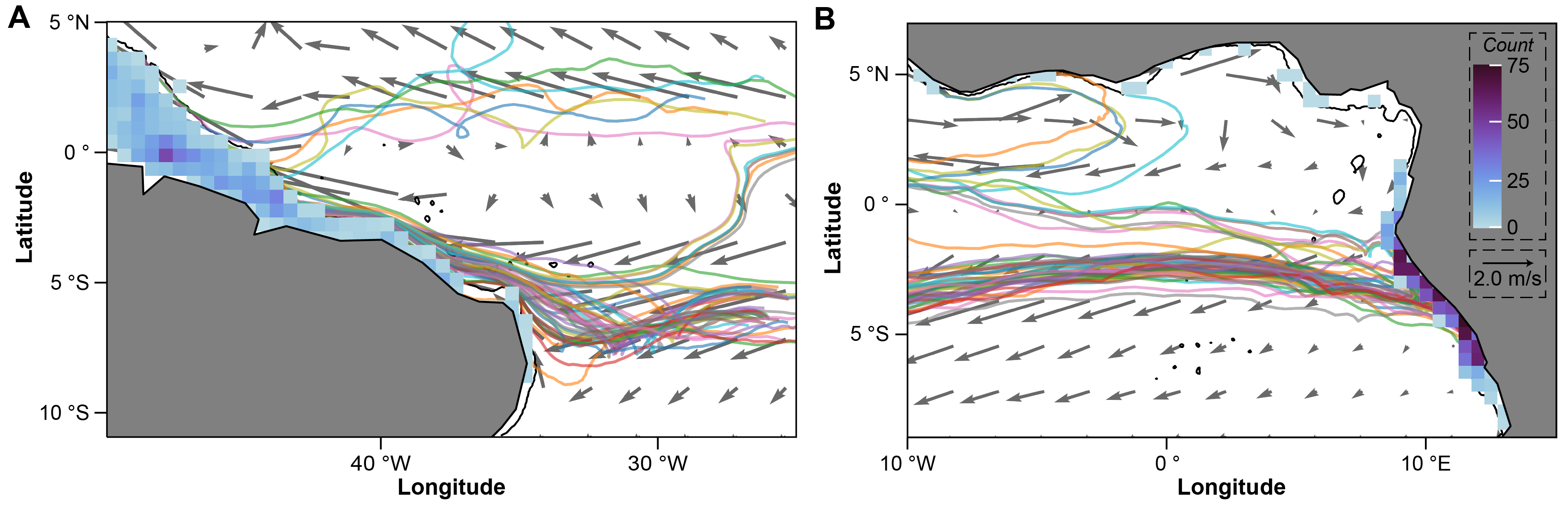

Supplement: S29 Fig — Distribution and abundance of particles that reach the coast of Brazil (A) and that depart from the coast of Africa (B) after 181 days of being released (i.e., light, with benefits from the light-dependent activity of chromoplast components). This is a zoomed-in version of Fig 4B. Grey arrows represent the 10-year mean velocity for the release depth (0–30 m) from VIKING20X. Every 50th arrow is shown. Black contours mark the 500 m isobath around Africa. Visualizations of larval dispersal used maps from cartopy [110], with the underlying vector map using NaturalEarth (naturalearthdata.com). (TIF) [file pbio.3003705.s029.tif]

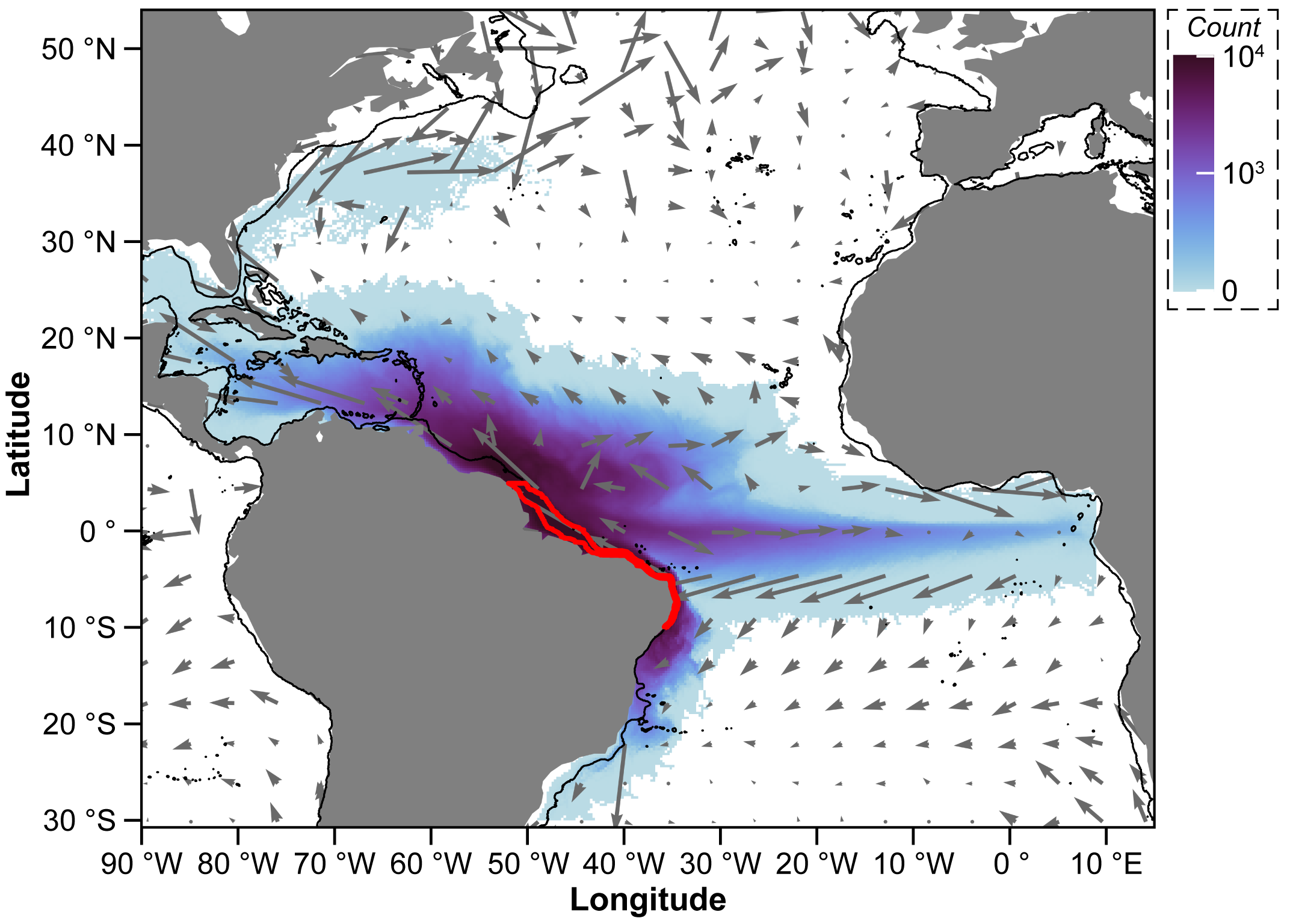

Supplement: S30 Fig — Distribution of particles in the Atlantic Ocean that were released along the northern coast of Brazil (red) after 181 (i.e., light, with benefits from the light-dependent activity of chromoplast components) days of being released. Grey arrows represent the 10-year mean velocity for the release depth (0–30 m) from VIKING20X. Every 100th arrow is shown. Black contours represent 500 m isobath. Visualizations of larval dispersal used maps from cartopy [110], with the underlying vector map using NaturalEarth (naturalearthdata.com). (TIF) [file pbio.3003705.s030.tif]

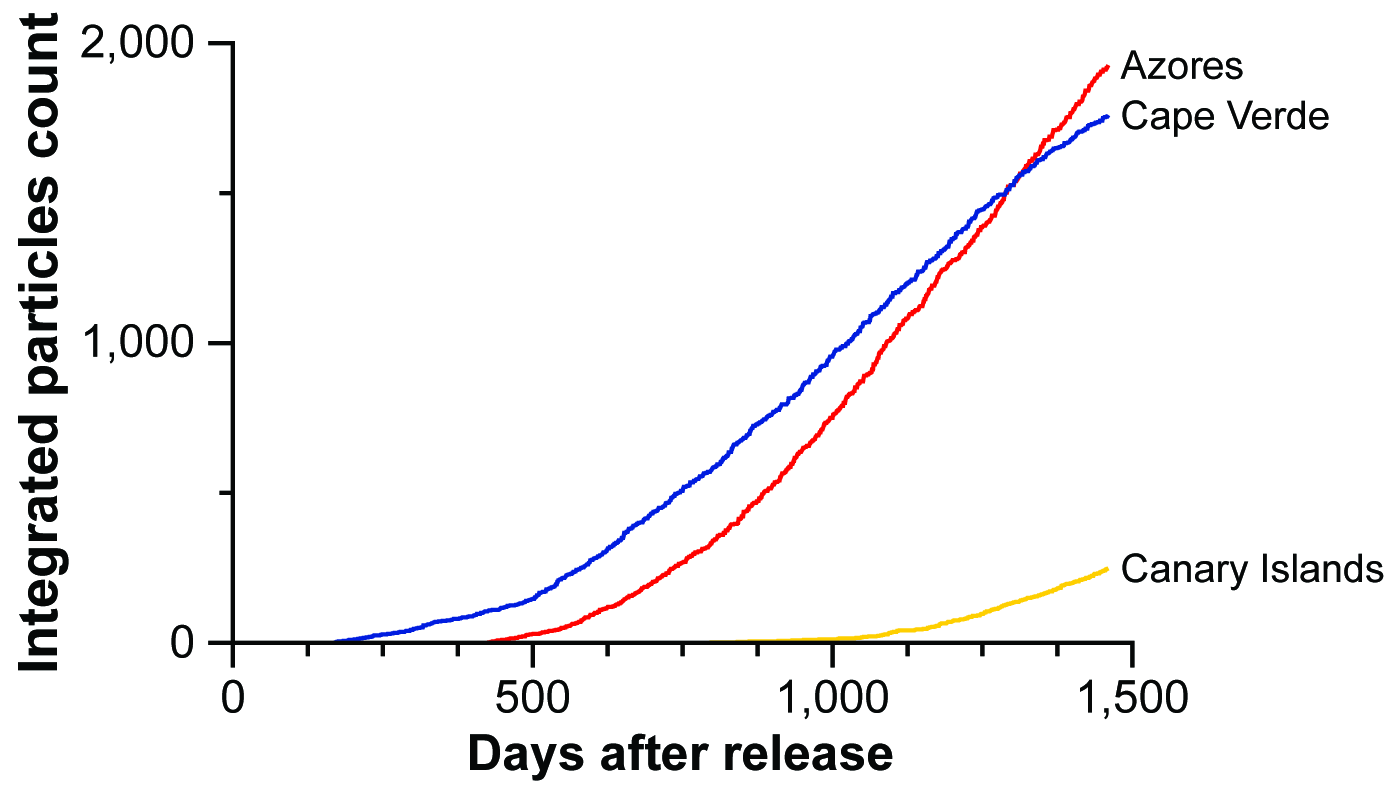

Supplement: S31 Fig — Duration required for particles to disperse from the northern coast of Brazil to Macaronesia. A particle was first detected in Cape Verde on day 170, the Azores on day 366, and the Canary Islands on day 783. This is based on the VIKING20X model and is the accumulation of annual releases from years 2007 to 2016. Corresponding raw data are presented in S15 Table. (TIF) [file pbio.3003705.s031.tif]

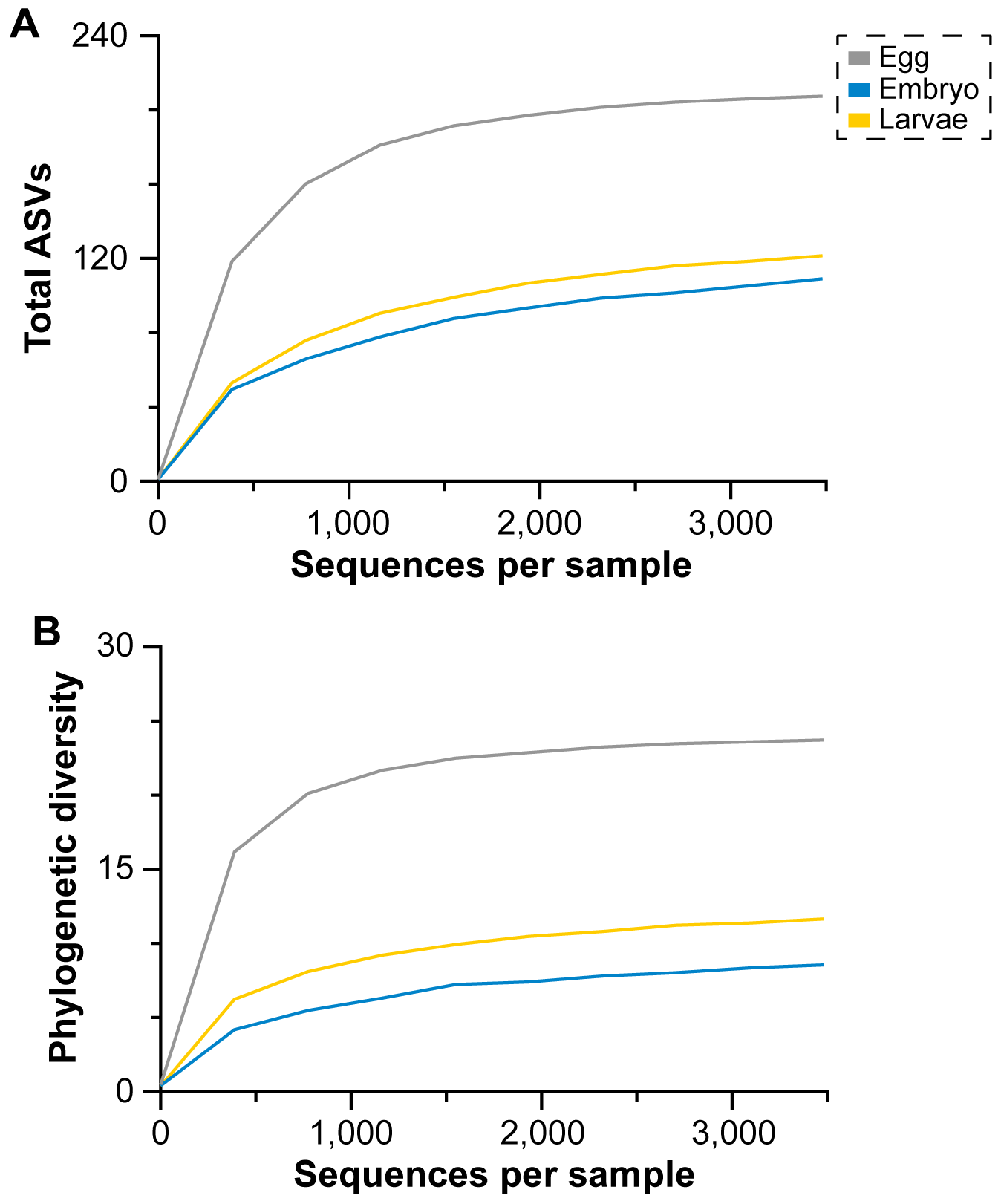

Supplement: S32 Fig — Rarefaction curves for the total ASVs (A) and phylogenetic diversity (B) of the microbial community associated with the developmental stages of the sea urchin Arbacia lixula. This was based on a rarefaction depth of 3,482 sequences and was used for all analyses. Corresponding raw data are presented in S16 Table. (TIF) [file pbio.3003705.s032.tif]

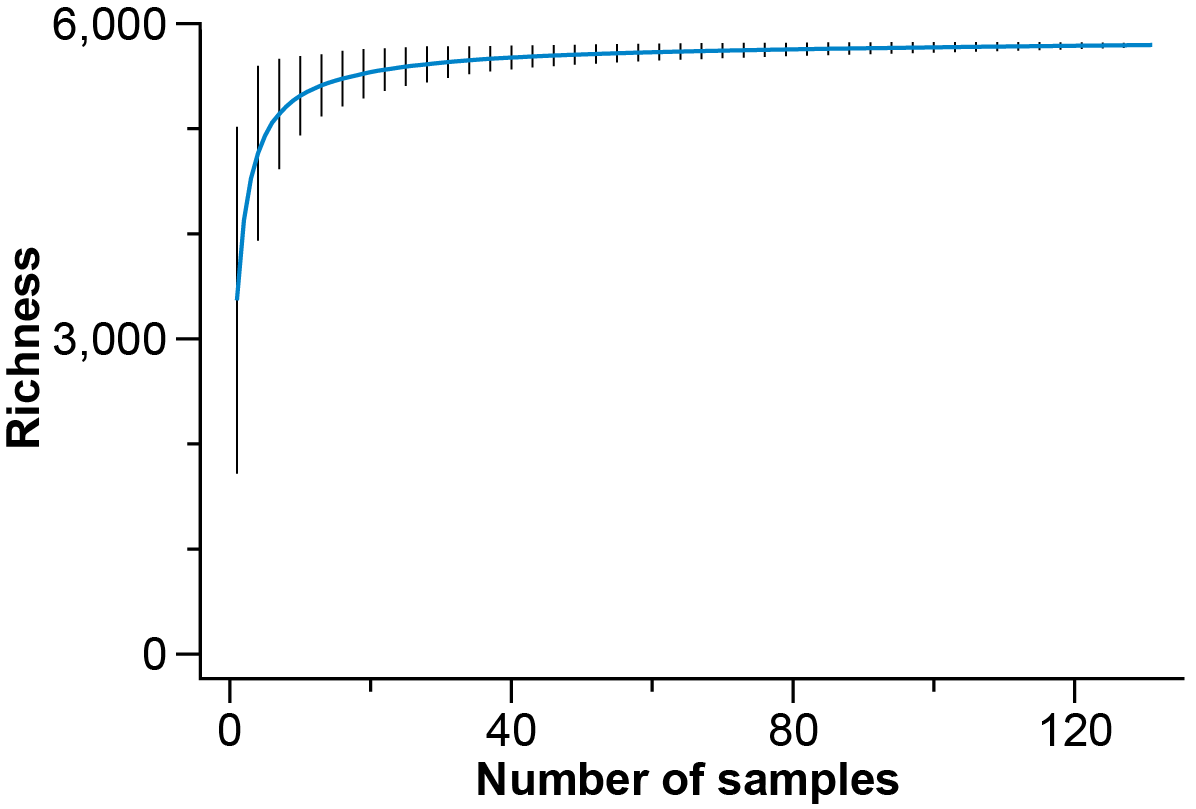

Supplement: S33 Fig — Rarefaction curve for the total metabolites observed based on the samples that were processed for the developmental stages of the sea urchin Arbacia lixula. (TIF) [file pbio.3003705.s033.tif]
